# Human endogenous retroviruses as epigenetic therapeutic targets in *TP53*-mutated diffuse large B-cell lymphoma

**DOI:** 10.1038/s41392-023-01626-x

**Published:** 2023-10-06

**Authors:** Ying Fang, Mu-Chen Zhang, Yang He, Chen Li, Hai Fang, Peng-Peng Xu, Shu Cheng, Yan Zhao, Yan Feng, Qian Liu, Li Wang, Wei-Li Zhao

**Affiliations:** 1 Shanghai Institute of Hematology, State Key Laboratory of Medical Genomics, National Research Center for Translational Medicine at Shanghai, Ruijin Hospital Affiliated to Shanghai Jiao Tong University School of Medicine, Shanghai, China; 2 Network and Information Center, Shanghai Jiao Tong University, Shanghai, China; 3 State Key Laboratory of Microbial Metabolism, School of Life Sciences and Biotechnology, Shanghai Jiao Tong University, Shanghai, China

**Keywords:** Haematological cancer, Tumour immunology

## Abstract

*TP53* mutation (*TP53*^mut^) occurs in 10–20% of diffuse large B-cell lymphoma (DLBCL) cases and serves as an unfavorable biomarker of DLBCL progression. It confers resistance to immunochemotherapy, high-dose chemotherapy, autologous stem cell transplantation, and anti-CD19 chimeric antigen receptor T-cell therapy. Therapeutic targeting of *TP53*^mut^ remains a significant challenge in DLBCL treatment. Here we assessed *TP53*^mut^ in 667 patients with newly diagnosed DLBCL, including 576 patients treated with immunochemotherapy rituximab, cyclophosphamide, doxorubicin, vincristine, and prednisone (R-CHOP) and 91 patients with decitabine plus R-CHOP (DR-CHOP, NCT02951728 and NCT04025593). *TP53*^mut^ independently predicted an inferior prognosis in R-CHOP-treated DLBCL, although this could be mitigated by DR-CHOP treatment. In *TP53*^mut^ patients, multiple viral regulation pathways were repressed, resulting in the inhibition of immune modulation, as revealed by gene set enrichment analysis. *TP53*^mut^ DLBCL exhibited increased methyltransferase *SUV39H1* expression and H3K9 trimethylation (H3K9me3), contributing to repression of endogenous retroviruses (ERVs) and immunosuppressive tumor microenvironment. In *TP53*^mut^ DLBCL cell lines, decitabine down-regulated *SUV39H1*, inhibited H3K9me3 occupancy on ERVs, and triggered ERV expression, thereby unleashing interferons program and CD4^+^T/CD8^+^T cell activation. Molecular silencing of *SUV39H1* significantly abrogated decitabine-induced H3K9me3 inhibition and ERV expression. In *TP53*^mut^ patient-derived xenograft models and *TP53*^mut^ patients, the anti-tumor effect was improved upon the use of combined treatment of decitabine and doxorubicin via SUV39H1-H3K9me3-ERVs axis. Collectively, our findings highlight an ERV regulatory circuitry in *TP53*^mut^ DLBCL and the crucial roles ERVs for epigenetically reprogramming tumor microenvironment for treating *TP53*^mut^-driven cancers.

## Introduction

Diffuse large B-cell lymphoma (DLBCL) represents the most common subtype of non-Hodgkin lymphoma and *TP53* mutation (*TP53*^mut^) is an important unfavorable genetic alteration,^1^ predominantly occurring in the DNA binding domain (DBD).^2^ Functionally, *TP53* mutants are classified as structural mutants (R175, R249, G245, and Y220) and DNA contact surface mutants (R248 and R273). Structural mutants reduce the thermostability of the protein, leading to improper folding at physiological temperatures and loss of DNA binding activity. DNA contact surface mutants are situated within the DNA core binding region and hinder the DNA-protein binding.^3^

*TP53*^mut^ confers resistance not only to conventional immunochemotherapy,^1,4,5^ but also to autologous stem-cell transplantation (ASCT),^6,7^ and chimeric antigen receptor T-cell (CAR-T) therapy in DLBCL,^8^ due to the immunosuppressive tumor microenvironment (TME), repression of interferon (IFN) response and inhibition of T-cell activation.^8,9^ The IFN family includes two main classes: type I and type II IFNs,^10^ both of which suppress tumors by directly acting on tumor cells and by indirectly enhancing anti-tumor immunity.^11,12^ Type I IFNs (mainly IFN-α and IFN-β) play a pivotal role in host defense against viral infection.^13^ Type II IFN (IFN-γ) is induced by IFN-α- or IFN-β-mediated activation of STAT4.^14^

5-Aza-2’-deoxycytidine (decitabine) is a hypomethylating agent that exhibits anti-tumor activity by reactivating tumor suppressor genes.^15^ In myelodysplastic syndrome (MDS) and acute myeloid leukemia (AML), the outcome of *TP53*^mut^ patients is significantly improved by decitabine treatment.^16,17^ Recently, we have reported a phase I/II trial of decitabine plus R-CHOP (DR-CHOP, NCT02951728) and found that all five *TP53*^mut^ patients achieve durable remission.^18^ Response to decitabine is related to a significant elevation of serum IFN-γ and increased peripheral blood CD3^+^CD4^+^T and CD3^+^CD8^+^T cells.^18^ However, the role of decitabine in reprogramming TME needs further investigation in *TP53*^mut^ DLBCL.

Human endogenous retroviruses (ERVs) are remnants of ancestral viral infections and permanently integrate within the human genome.^19^ DNA methylation and trimethylation of histone H3 on lysine 9 (H3K9me3) are important epigenetic mechanisms in modulating ERV expression,^20^ inhibiting IFN-γ production, and impairing T-cell activation.^21^ DNA methyltransferase 1 (DNMT1) is the main factor inducing DNA methylation; meanwhile, the depletion of DNMT1 leads to ERV de-repression.^22^ Decitabine induces DNA demethylation through DNMT1 depletion and restores ERV transcription, provoking the anti-tumor activity.^23^ SUV39 family and PRDM family members function as the main methyltransferases of H3K9.^24^ SUV39 family members repress ERV transcription through H3K9 methylation.^21,25^ Whether H3K9 methylation can be targeted by decitabine remains of great interest. The viral mimicry anti-tumor effects exerted by ERVs also command great attention.

Here we first explored the pivotal role of *TP53*^mut^ in modulating the ERV expression and the TME reprogramming in a histone methylation-dependent manner. Then we illustrated the underlying mechanism of decitabine on ERV regulatory circuitry and anti-tumor immunity in *TP53*^mut^ DLBCL. Our findings thus provide both in vitro and in vivo evidence that ERV functions as a new epigenetic therapeutic target in *TP53*^mut^-driven cancers.

## Results

### *TP53*^mut^ contributes to tumor progression in patients with DLBCL

**Fig. 1:**
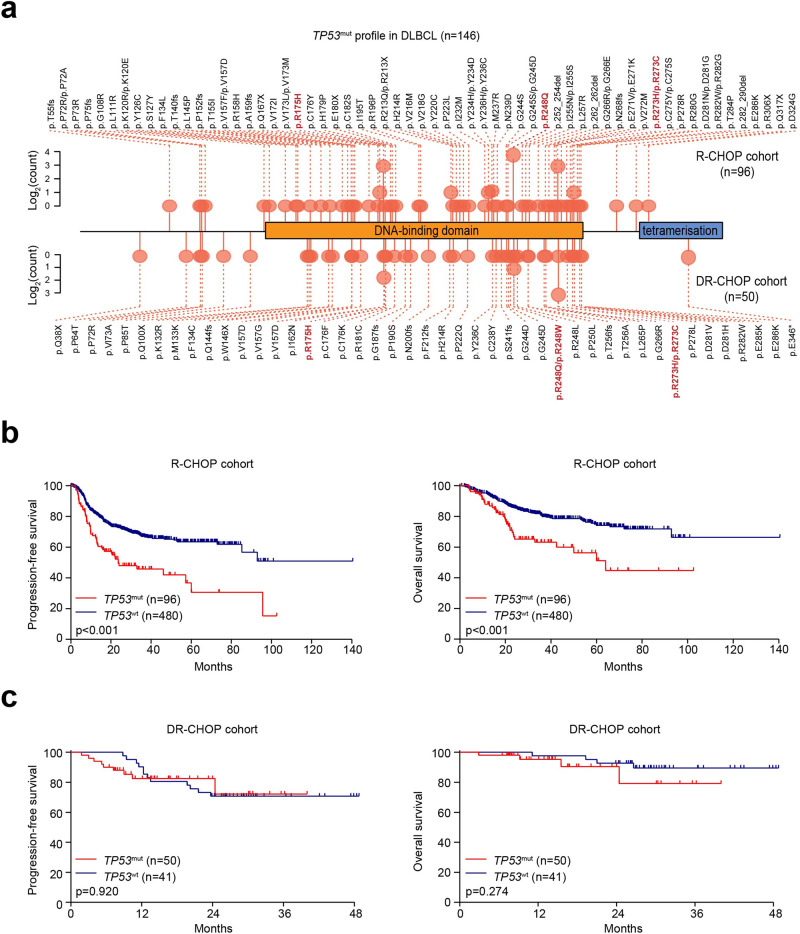
Mutational profile and survival analysis of patients with *TP53*^*mut*^ DLBCL. **a** Mutational profile of *TP53*^mut^ DLBCL patients. **b**, **c** PFS and OS of patients upon R-CHOP (**b**) or DR-CHOP (**c**) treatment according to *TP53*^mut^ status

A flow chart describing the selection of cohorts was outlined in Supplementary Fig. 1. Among 667 DLBCL patients analyzed in this study, 146 patients had *TP53*^mut^ (125 nonsynonymous single nucleotide variant, 7 frameshift deletion, 9 stop-gain, 3 frameshift insertion, 2 splicing, 1 non-frameshift insertion, 1 non-frameshift deletion and 2 non-frameshift substitution, Fig. 1a), in which 133 patients (91.1%) had mutations located in DBD. Regarding treatment, 576 patients (96 with *TP53*^mut^ and 480 with *TP53*^wt^) received R-CHOP, and 91 patients (50 with *TP53*^mut^ and 41 with *TP53*^wt^) received DR-CHOP.

As shown in Supplementary Table 1, *TP53*^mut^ was significantly associated with elevated serum lactate dehydrogenase (LDH) levels (*p* = 0.013). Among 576 patients of the R-CHOP cohort, with a median follow-up of 31.4 months (range 1.0–140.5), *TP53*^mut^ patients had shorter survival time than *TP53*^wt^ patients, with 3-year progression-free survival (PFS) of 46.1% (95%CI 34.8–56.6) vs. 67.1% (95%CI 62.3–71.4, *p* < 0.001) and 3-year overall survival (OS) of 65.1% (95%CI 53.1–74.8) vs. 80.6% (95%CI 76.3–84.2) (*p* < 0.001, Fig. 1b). While in the DR-CHOP cohort, with a median follow-up of 24.5 months (range 2.9–48.8), *TP53*^mut^ patients and *TP53*^wt^ patients had similar 3-year PFS [72.1% (95%CI 44.6–87.6) vs. 70.7% (95%CI 54.3–82.2), *p* = 0.920] and 3-year OS [79.1% (95%CI 44.7–93.4) vs. 89.5% (95%CI 74.1–96.0), *p* = 0.274, Fig. 1c].

Age, sex, ECOG performance status, Ann Arbor stage, serum LDH level, extranodal involvement, and *TP53* mutational status were included in univariate analysis. Variables demonstrating significance with *p* < 0.200 on univariate analysis were included in the multivariate model.^26^ Univariate analysis showed that age >60 years, ECOG ≥ 2, Ann Arbor stage III/IV, elevated serum LDH, multiple extranodal involvement, and *TP53*^mut^ were unfavorable prognostic indicators for both PFS and OS in the R-CHOP cohort. By multivariate analysis, Ann Arbor stage III/IV, elevated serum LDH, and *TP53*^mut^ were independent factors predicting poor PFS and OS (all *p* ≤ 0.001) (Supplementary Table 2). However, in the DR-CHOP cohort, univariate analysis showed only ECOG ≥ 2 as an adverse prognostic factor for both PFS and OS (*p* = 0.017 and 0.027, respectively). By multivariate analysis, ECOG ≥ 2 was an independent factor predicting poor PFS and OS (*p* = 0.037 and 0.013, respectively) (Supplementary Table 3).

### *TP53*^mut^ is associated with ERV down-regulation and immunosuppressive TME in DLBCL

**Fig. 2:**
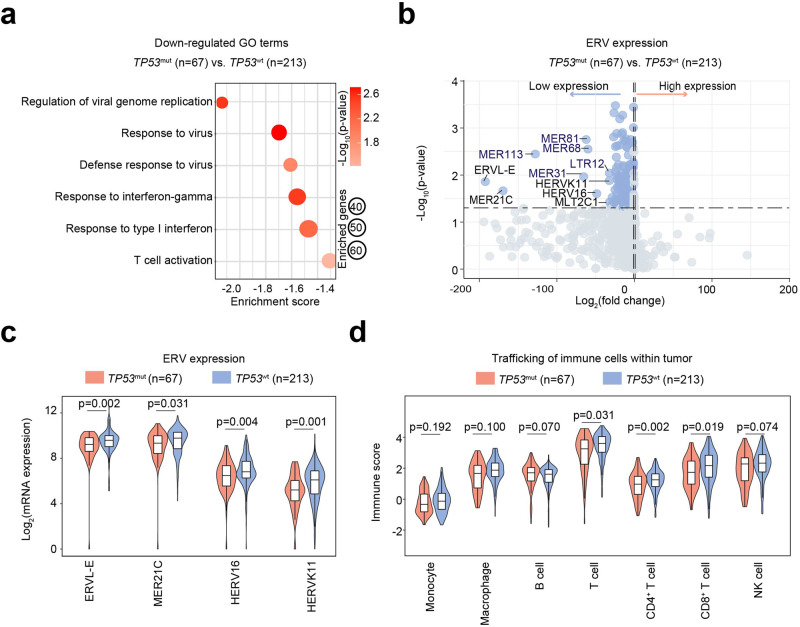
ERV repression and immunosuppressive TME in *TP53*^mut^ DLBCL. **a** Down-regulated gene ontology (GO) terms in *TP53*^mut^ DLBCL patients, as compared to *TP53*^wt^ DLBCL patients. The color of points indicates the -log (p-value) of dysregulated pathways in two groups. The size of points indicates the number of genes included in each gene set. **b** Volcano plot image of the ERV expression in *TP53*^mut^ DLBCL and *TP53*^wt^ DLBCL. The gray dashed line corresponds to *p* = 0.05. The significantly depressed ERVs in *TP53*^mut^ DLBCL are annotated. **c** ERV expression in DLBCL according to *TP53*^mut^ status. **d** Immunity activity scores of indicated immune cells in *TP53*^mut^ DLBCL and *TP53*^wt^ DLBCL. P-values comparing different scores in two groups. *TP53*^mut^, mutant *TP53*; *TP53*^wt^, wild-type *TP53*

To identify the potential role of *TP53*^mut^ on the TME of DLBCL, we performed RNA sequencing on 280 *TP53*^mut^ DLBCL (67 with *TP53*^mut^ and 213 with *TP53*^wt^). Interestingly, in *TP53*^mut^ patients, in addition to activated pathways involved in *TP53*-targeted biological functions such as positive regulation of cell cycle and negative regulation of intrinsic apoptosis (Supplementary Fig. 2a), multiple viral regulation pathways (regulation of viral genome replication, response to virus, and defense response to virus) were shown to be repressed, accompanied by the inhibition of immune modulation (response to IFN-gamma, response to type I IFN, and T cell activation), as revealed by gene set enrichment analysis (GSEA) (Fig. 2a).

To further determine the potential virus involved in *TP53*^mut^ DLBCL, viral sequences were assessed and quantified by the Kraken 2^27^ and ERVs were assessed and quantified by RepeatMasker (hg19) based on the database downloaded from Repeating Elements. Among the main virus genera detected, only ERVs were remarkably down-regulated in *TP53*^mut^ DLBCL (Supplementary Table 4). The volcano plot showed significantly lower expression levels of multiple ERVs in *TP53*^mut^ than in *TP53*^wt^ tumors (Fig. 2b). ERVL-E, MER21C, HERV16, and HERVK11 were the most significantly down-regulated ERVs (Fig. 2c). Meanwhile, tracking tumor immunophenotype (TIP) analysis indicated that the recruitment activities of T cells (*p* = 0.031), CD4^+^T (*p* = 0.002), and CD8^+^T cells (*p* = 0.019) were significantly decreased in *TP53*^mut^ DLBCL, as compared to the *TP53*^wt^ DLBCL (Fig. 2d). Consistent with the lower infiltration rate of T cells, the enriched genes involved in MHC-I and MHC-II protein complex, as well as IFN-stimulated genes, were significantly repressed in *TP53*^mut^ DLBCL (Supplementary Fig. 2b).

### *TP53*^mut^ down-regulates ERV expression through enhancing SUV39H1 expression and H3K9 trimethylation

**Fig. 3:**
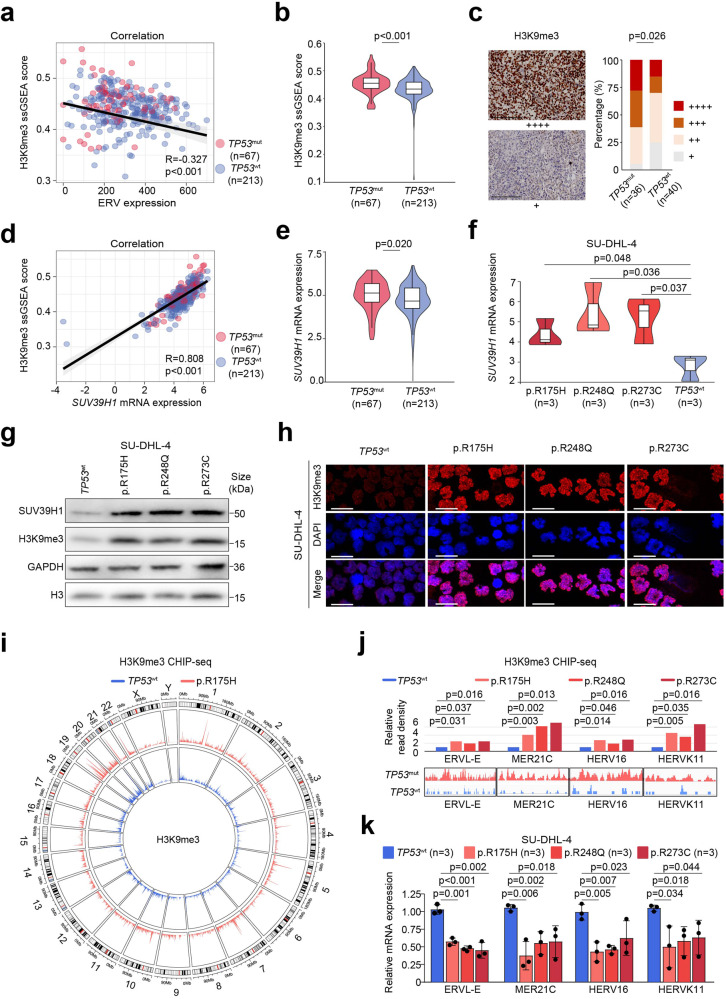
H3K9me3 enrichment and ERV expression in *TP53*^mut^ DLBCL. **a** Correlations of the H3K9me3 score with ERV expression. **b** H3K9me3 score in DLBCL patients according to *TP53*^mut^ status. **c** Immunohistochemistry staining of H3K9me3 in DLBCL according to *TP53*^mut^ status. Left panel, representative immunohistochemistry staining for H3K9me3. Right panel, proportion of H3K9me3 expression. Scale bars, 100 μm. Expression levels were assessed according to the percentage of positive cells: + denoted <25%; ++ denoted 25–49%; +++ denoted 50–74%; ++++ denoted 75–100%. **d** Correlations of *SUV39H1* expression with the H3K9me3 score. **e** *SUV39H1* expression according to RNA-sequencing data of lymphoma samples from *TP53*^mut^ DLBCL and *TP53*^wt^ DLBCL. **f** *SUV39H1* expression in *TP53*^mut^ or *TP53*^wt^ SU-DHL-4 cell line. **g** H3K9me3 expression in *TP53*^mut^ or *TP53*^wt^ SU-DHL-4 cell line by western blot. **h** H3K9me3 expression in *TP53*^mut^ or *TP53*^wt^ SU-DHL-4 cell line by immunofluorescence staining. Scale bars, 40 μm. **i** Distribution of H3K9me3 on the whole genome in *TP53*^mut^ p.R175H or *TP53*^wt^ SU-DHL-4 cell line. **j** H3K9me3 signal profiles in the genomic regions of ERVs in *TP53*^mut^ or *TP53*^wt^ SU-DHL-4 cell line. Upper, relative enrichment of H3K9me3 domains at the indicated ERVs; lower, the genome browser view of H3K9me3 signals on the indicated ERVs. Signals represent ChIP-seq RPM. **k** Expression of ERVs in *TP53*^mut^ or *TP53*^wt^ SU-DHL-4 cell line

Since methylation status controls ERV expression, we analyzed the relationship between different methylation scores^28^ with ERV levels. As shown in Fig. 3a, only the H3K9me3 score was negatively correlated with ERV expression in DLBCL patients (*p* < 0.001), while no significant correlation was observed among other methylation processes and ERV levels (Supplementary Fig. 3a). H3K9me3 score was significantly increased (*p* < 0.001, Fig. 3b), with H3K9me3 protein expression increased by immunohistochemistry in *TP53*^mut^ DLBCL (*p* = 0.026, Fig. 3c). Among major H3K9 lysine methyltransferases [including SUV39 family (SUV39H1, SUV39H2, SETDB1, SETDB2, G9a, and GLP) and PRDM family members (PRDM2, PRDM3, PRDM8, and PRDM16)],^24^ only *SUV39H1* expression was positively correlated with H3K9me3 score (*p* < 0.001, Fig. 3d and Supplementary Fig. 3b) and significantly higher in *TP53*^mut^ DLBCL than in *TP53*^wt^ DLBCL patients (*p* = 0.020, Fig. 3e), with SUV39H1 protein expression increased by immunohistochemistry in *TP53*^mut^ DLBCL (*p* = 0.043, Supplementary Fig. 3c).

To explore the underlying mechanisms of *TP53*^mut^ on ERV expressions, we established *TP53*^mut^ (p.R175H, p.R248Q, p.R273C) cell lines in both GCB (SU-DHL-4) and ABC (OCI-LY-3) subtypes. Representative structural mutant R175H, as well as DNA contact surface mutants R248Q and R273C, were used in our experimental models to illustrate the aberrant function of mutant *TP53*. In both SU-DHL-4 (Fig. 3f) and OCI-LY-3 cells (Supplementary Fig. 4a), expression of *SUV39H1* was higher in *TP53*^mut^ cells than in *TP53*^wt^ cells. Comparable to the gene expression, the high protein expression of SUV39H1 was confirmed by western blot (Fig. 3g and Supplementary Fig. 4b) and immunofluorescence staining (Supplementary Fig. 4c). As per the literature review, the expression of E2F1 is up-regulated in *TP53*-knockdown cells.^29^ In our study, E2F1 expression levels were significantly higher in *TP53*^mut^ DLBCL patients (Supplementary Fig. 4d) and *TP53*^mut^ cell lines (Supplementary Fig. 4e and 4f) than *TP53*^wt^ counterparts. According to the human transcription factor target database,^30^ E2F1 could bind to the promoter of *SUV39H1*. Luciferase-reporter assay was then performed, and the results confirmed that E2F1 could directly activate *SUV39H1* through transcriptional regulation (Supplementary Fig. 4g). As the results of elevated expression of SUV39H1, H3K9me3 levels were significantly increased in *TP53*^mut^ cells by western blot (Fig. 3g and Supplementary Fig. 4b) and immunofluorescence staining (Fig. 3h).

Molecular silencing of *SUV39H1* was induced by transfecting with *SUV39H1* short hairpin RNA (shRNA) in *TP53*^mut^ SU-DHL-4 and OCI-LY-3 cell lines, and vector, as well as non-targeting shRNA, were used as control (Supplementary Fig. 5a, b). Lack of *SUV39H1* expression resulted in a significant decrease of H3K9me3 (Supplementary Fig. 5b) and an increase in ERV expression (Supplementary Fig. 5c), indicating that ERV repression was dependent on SUV39H1 expression in *TP53*^mut^ DLBCL. As a consequence of ERV activation, remarkable up-regulation of IFN (Supplementary Fig. 5d) and increased CD4^+^T, CD8^+^T cells (Supplementary Fig. 5e) were observed in *SUV39H1*-shRNA cells, as compared to control cells.

ChIP-seq was further performed to investigate the genomic distribution of H3K9me3 and revealed a significant enrichment of H3K9me3 on the whole genome in *TP53*^mut^ cells, as compared to *TP53*^wt^ cells (Fig. 3i and Supplementary Fig. 6a). Of note, H3K9me3 was significantly enriched on the sequence of ERVL-E, MER21C, HERV16, and HERVK1 (Fig. 3j), resulting in repression of these ERVs in *TP53*^mut^ SU-DHL-4 and OCI-LY-3 cells (Fig. 3k and Supplementary Fig. 6b). Consequently, concentrations of IFN [IFN-α, (*p* = 0.043), IFN-β (*p* = 0.015), and IFN-γ (*p* < 0.001)] were lower in the supernatants of *TP53*^mut^ cells than in *TP53*^wt^ cells (Supplementary Fig. 7a). To validate the influence of *TP53*^mut^ on TME, *TP53*^mut^ cells were co-cultured with peripheral blood mononuclear cells (PBMCs). *TP53*^mut^ cells inhibited the growth of CD4^+^T and CD8^+^T cells, as compared to *TP53*^wt^ cells (Supplementary Fig. 7b). Since DNMT1 was reported to play a crucial role in ERV repression, we evaluated the expression of DNMT1. However, no significant difference was observed between *TP53*^mut^ and *TP53*^wt^ DLBCL patients (Supplementary Fig. 8a) and *TP53*^wt^ cell lines (Supplementary Fig. 8b–8d), suggesting DNMT1 may not be involved in regulating ERVs in *TP53*^mut^ DLBCL.

### Decitabine combined with doxorubicin enhances ERV activation in *TP53*^mut^ DLBCL both in vitro and in vivo via SUV39H1-H3K9me3 axis

**Fig. 4:**
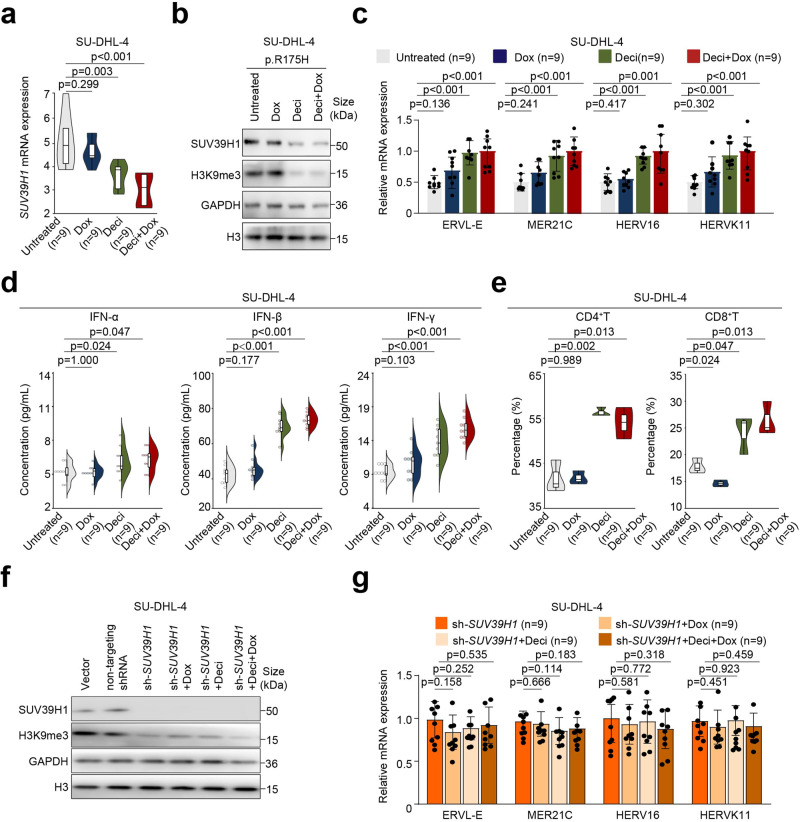
Decitabine combined with doxorubicin on ERV activation in *TP53*^mut^ SU-DHL-4 cells. **a** *SUV39H1* expression detected by qRT-PCR in *TP53*^mut^ SU-DHL-4 cell line treated with decitabine, doxorubicin, alone or in combination. **b** The expression of SUV39H1 and H3K9me3 detected by western blot in *TP53*^mut^ SU-DHL-4 cell line upon indicated treatment group. **c**–**e** ERV expression (**c**), IFN production (**d**), as well as percentages of CD4^+^T and CD8^+^T cells (**e**) upon indicated treatment group. **f** Expression of SUV39H1 and H3K9me3 in *SUV39H1* knock-down SU-DHL-4 cell line upon indicated treatment group. **g** ERV expression in *SUV39H1* knock-down SU-DHL-4 cell line upon indicated treatment group

Both SU-DHL-4 and OCI-LY-3 *TP53*^mut^ (p.R175H, p.248Q, p.R273C) cells were exposed to decitabine (deci, 330 nM) alone, doxorubicin (dox, key cytotoxic agent of R-CHOP, 200 nM) alone, and combined treatment (deci+dox, deci 330 nM for 5 days followed by dox 200 nM for 2 days, based on clinically achievable concentrations). Compared with untreated cells, decitabine combined with doxorubicin significantly down-regulated *SUV39H1* and H3K9me3 expression in *TP53*^mut^ SU-DHL-4 (Fig. 4a, b, Supplementary Fig. 9a–c) and OCI-LY-3 cells (Supplementary Fig. 9a). In response to the down-regulation of H3K9me3, expression levels of ERVs were markedly increased (Fig. 4c and Supplementary Fig. 9d), along with increased concentrations of IFN-α (*p* = 0.047), IFN-β (*p* < 0.001), and IFN-γ (*p* < 0.001) in the supernatant of *TP53*^mut^ cells when treated with decitabine and doxorubicin (Fig. 4d and Supplementary Fig. 9e). When *TP53*^mut^ cells were co-cultured with PBMCs, CD4^+^T and CD8^+^T cells were significantly increased upon combined treatment (Fig. 4e and Supplementary Fig. 9f). However, in *TP53*^mut^ cells transfected with *SUV39H1* shRNA, the effect of decitabine and doxorubicin on SUV39H1, H3K9me3 (Fig. 4f, Supplementary Fig. 10a, b) and ERV expression (Fig. 4g and Supplementary Fig. 10c) were abrogated, reinforcing the essential role of SUV39H1 in epigenetic reprogramming upon combined treatment. Of note, the sequences of ERVL-E, MER21C, HERV16, and HERVK11 were mainly unmethylated, as revealed by the bisulfite-PCR assay (Supplementary Fig. 11), indicating a limited role of DNA methylation in the regulation of ERV expression in *TP53*^mut^ DLBCL.

**Fig. 5:**
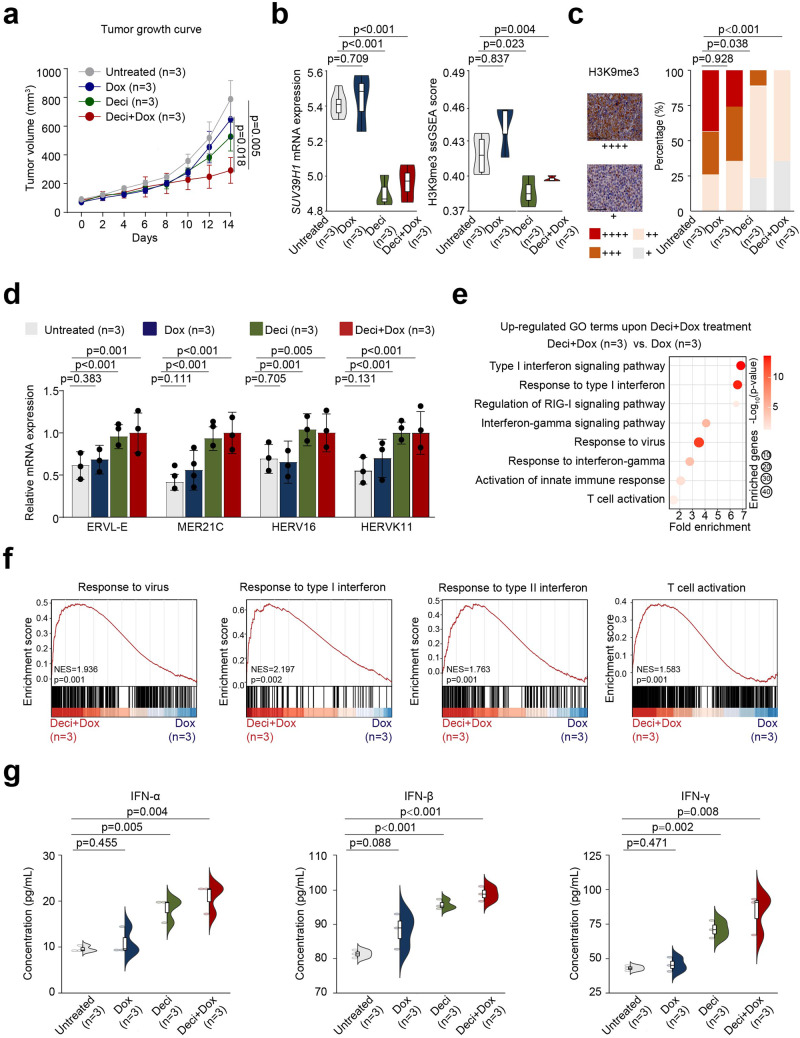
Decitabine combined with doxorubicin on ERV activation, IFN production, and anti-tumor effect in *TP53*^*mut*^ PDX models. **a** Decitabine and doxorubicin treatment significantly retarded tumor growth of *TP53*^mut^ PDX models, as compared to untreated and single treatment groups. **b** Decitabine and doxorubicin down-regulated *SUV39H1* expression and H3K9me3 score in *TP53*^mut^ tumors. **c** Decreased H3K9me3 expression revealed by immunohistochemistry staining on *TP53*^mut^ tumors. Left panel, representative immunohistochemistry staining images for H3K9me3. Right panel, proportion of H3K9me3 expression. Scale bars, 50 μm. Expression levels were assessed according to the percentage of positive cells: + denoted <25%; ++ denoted 25–49%; +++ denoted 50–74%; ++++ denoted 75–100%. **d** High expression of ERVs in lysed *TP53*^mut^ PDX tumors treated with decitabine and doxorubicin, as compared to untreated and single treatment groups. **e** Up-regulated gene ontology (GO) terms in *TP53*^mut^ tumors treated with decitabine combined with doxorubicin, as compared to single doxorubicin treatment. **f** Significantly activated signaling pathways identified by RNA-seq in *TP53*^mut^ tumors treated with decitabine combined with doxorubicin, as compared to single doxorubicin treatment. Enrichment plots of response to virus signaling pathway, type I/type II interferon signaling pathway, and T cell activation involved in immune response pathway by GSEA analysis. **g** The concentrations of IFN-α, IFN-β, and IFN-γ in *TP53*^mut^ tumors treated with decitabine and doxorubicin, as compared to untreated and single treatment groups

To validate in vitro effect of decitabine and doxorubicin in *TP53*^mut^ DLBCL, patient-derived xenograft models (PDX, *TP53*^mut^ p.R175H, p.R248Q and p.R273C) were established and treated with doxorubicin, either alone or combined with decitabine. At day 14, decitabine and doxorubicin significantly retarded tumor growth, as compared to untreated group (tumor volume: 291.9 ± 73.0 mm^3^ vs. 788.9 ± 106.0 mm^3^, *p* = 0.005, Fig. 5a) and doxorubicin treated group (tumor volume: 291.9 ± 73.0 mm^3^ vs. 647.7 ± 107.1 mm^3^, *p* = 0.018, Fig. 5a), respectively. As a mechanism of action, combined treatment significantly down-regulated *SUV39H1* expression, H3K9me3 score (Fig. 5b and Supplementary Fig. 12a), and H3K9me3 expression (Fig. 5c) in *TP53*^mut^ PDX tumors. Correspondingly, ERVs expression levels were prominently elevated in *TP53*^mut^ PDX models for the combined treatment group as compared to the untreated group [ERVL-E (1.0 ± 0.2 vs. 0.6 ± 0.2, *p* = 0.001), MER21C (1.0 ± 0.2 vs. 0.4 ± 0.1, *p* < 0.001), HERV16 (1.0 ± 0.2 vs. 0.7 ± 0.2, *p* = 0.005), and HERVK11 (1.0 ± 0.2 vs. 0.6 ± 0.1, *p* < 0.001)] (Fig. 5d). Using GO analysis, multiple signaling pathways were significantly up-regulated in *TP53*^mut^ PDX tumors upon combined treatment, such as response to the virus, IFN pathways (type I IFN signaling pathway, response to type I IFN, positive regulation of RIG-I signaling pathway, IFN-γ-mediated signaling pathway, and response to IFN-γ), as well as immune modulation (activation of innate immune response and T cell activation) (Fig. 5e, 5f). Consequently, the levels of IFN-α, IFN-β, and IFN-γ in PDX tumors showed a significant increase in both the decitabine group and the combined treatment group, when compared to the untreated group (Fig. 5g).

### DR-CHOP-responding *TP53*^mut^ DLBCL patients show reprogramming of TME and restoration of anti-tumor immunity

**Fig. 6:**
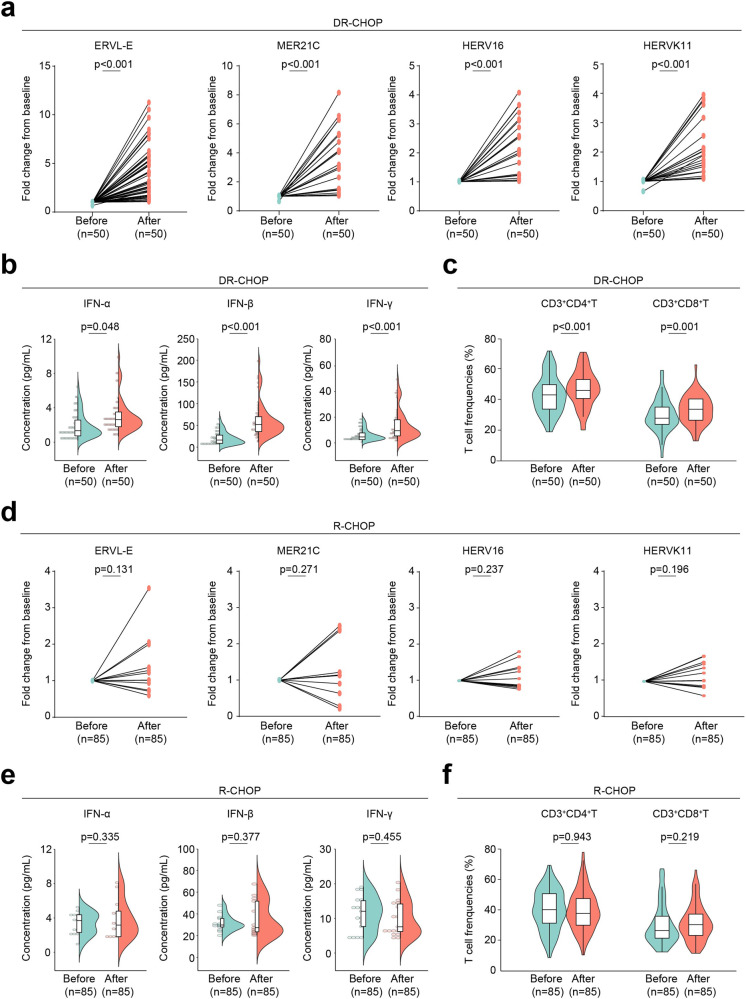
Induction of ERV expression and IFN production upon DR-CHOP or R-CHOP treatment in DLBCL patients. **a**–**c** Increased ERV expression (**a**), IFN production (**b**), as well as percentages of CD3^+^CD4^+^T and CD3^+^CD8^+^T cells (**c**) in *TP53*^mut^ patients after one cycle of DR-CHOP. **d**–**f** ERV expression (**d**), IFN production (**e**), as well as percentages of CD3^+^CD4^+^T and CD3^+^CD8^+^T cells (**f**) in *TP53*^mut^ patients after one cycle of R-CHOP

Expression levels of ERVs were increased in the plasma of *TP53*^mut^ patients after the first cycle of DR-CHOP treatment (*p* all <0.001, Fig. 6a), accompanied by increased serum concentrations of IFN-α (*p* = 0.048), IFN-β (*p* < 0.001), IFN-γ (*p* < 0.001, Fig. 6b), and elevated frequencies of CD3^+^CD4^+^T (*p* < 0.001) and CD3^+^CD8^+^T cells (*p* = 0.001, Fig. 6c) in the DR-CHOP-responding patients with *TP53*^mut^ DLBCL. On the contrary, up-regulation of ERVs (Fig. 6d), as well as IFN production (Fig. 6e) and T cell activation (Fig. 6f), were not observed among *TP53*^mut^ patients after the first cycle of R-CHOP, highlighting the essential role of epigenetic activation of ERVs in *TP53*^mut^ DLBCL. Together, decitabine combined with doxorubicin reprogrammed TME by enhancing ERV expression, unleashing the IFN program, promoting T-cell activation, and improving the outcome of *TP53*^mut^ DLBCL.

## Discussion

*TP53*^mut^ confers immunochemotherapy resistance and poor clinical outcome of DLBCL patients.^1,6–8^ As an alternative mechanism of lymphoma progression, *TP53*^mut^ alters host innate and adaptive immune responses,^9^ thus contributing to immunosuppressive TME, with decreasing CD4^+^T and CD8^+^T cell components.^8,9^ To our knowledge, this is the first report to identify ERVs as *TP53*^mut^-downstream targets in DLBCL, whose inhibition depended on a histone-methylation manner, resulting from increased SUV39H1 expression and H3K9 methylation. Consistently, molecular silencing of *TP53* in solid tumor cells increases SUV39H1 expression and induces H3K9me3, leading to cell cycle progression and chemoresistance.^29,31,32^ Of note, as revealed by Chip-seq, *TP53*^mut^ mediated enrichment of H3K9me3 for whole genome and H3K9me3 deposition sites on ERVs are at least part of the H3K9 epigenetic program to regulate H3K9me3-target genes. Therefore, the genome-wide epigenetic down-regulation by *TP53*^mut^ was associated with ERV repression and consequent IFN inhibition, contributing to impaired anti-tumor immunity in *TP53*^mut^ DLBCL. Our study not only proposed a functional role of *TP53*^mut^ on TME within DLBCL, but also provided a potential rationale to overcome the adverse effect of *TP53*^mut^ in DLBCL progression.

Activation of TP53 induces viral mimicry response, unleashes the interferon program, and increases CD4^+^T and CD8^+^T cell proportion, raising possibilities of targeting ERVs as an anti-tumor approach since directly targeting *TP53*^mut^ is challenging.^33^ Moreover, we were inspired by our previous study, in which DR-CHOP-responding *TP53*^mut^ DLBCL patients have elevated serum IFN concentrations and increased proportions of peripheral blood CD3^+^CD4^+^T and CD3^+^CD8^+^T cells.^18^ In this study, we confirmed our previous results that DR-CHOP improved the outcome of *TP53*^mut^ DLBCL patients and further elucidated the underlying mechanism of DR-CHOP on epigenetic regulation through modulating ERV expression, IFN production, and T cell activation in *TP53*^mut^ DLBCL. Mutant *TP53* shapes the immune landscape of the TME by down-regulating MHC-1/II expression and T cell infiltration,^9^ decitabine treatment increased ERV expression, IFN production, and mediated viral mimicry anti-tumor immunity, which is why, *TP53*^mut^ DLBCL, resistant to R-CHOP, could be eliminated by decitabine combined with chemotherapy (DR-CHOP). Consistently, in MDS and AML, decitabine induces higher IFN production^34^ and improves responses in *TP53*^mut^ patients.^35,36^ These observations demonstrated that reprogramming TME was essentially involved in decitabine-treated *TP53*^mut^ hematological malignancies through fostering IFN production and T cell activation.

Axis of virus-IFN-T-cell activation is a well-known mechanism in anti-virus treatment. Growing preclinical and clinical evidence suggests that hypomethylating agents can induce DNA demethylation and ERV reactivation, resulting in a viral mimicry state to generate anti-tumor immune responses.^18,37^ However, the expression of DNMT1 showed no significant difference between *TP53*^mut^ and *TP53*^*w*t^ DLBCL patients and cell lines. Furthermore, the bisulfite-PCR analysis showed that the sequences of ERVL-E, MER21C, HERV16, and HERVK11 were mainly unmethylated, and decitabine failed to change the unmethylated status of these ERVs, indicating that DNA methylation was less involved in *TP53*^mut^ DLBCL. Consistent with our results, in mouse embryonic stem cells, 69 ERV subfamilies were de-repressed in the SETDB1 knockout cells, and only 5 were de-repressed in the DNMT1/DNMT3A/DNMT3B triple knockout cells, indicating that the majority of ERVs were controlled by histone methylation rather than DNA methylation.^38^ Indeed, in *TP53*^mut^ DLBCL cell lines and PDX murine models, we illustrated that decitabine induced ERV expression through the down-regulation of SUV39H1 and inhibition of H3K9me3. Furthermore, molecular silencing of *SUV39H1* abrogated the effects of decitabine and doxorubicin on H3K9me3 and ERV expressions, underlining the pivotal role of SUV39H1 in epigenetic reprogramming upon decitabine treatment. This is consistent with solid tumors in which decitabine impairs SUV39H1 recruitment and inhibits H3K9me3, thereby blocking tumor cell growth and metastasis.^39^

It is also worth pointing out that upon DR-CHOP treatment, *TP53*^mut^ DLBCL obtained 3-year PFS and 3-year OS with 72.1% and 79.1%, respectively, similar to *TP53*^wt^ DLBCL that received R-CHOP.^7^ Collectively, our findings highlighted a TP53/SUV39H1/H3K9me3 regulatory circuitry and assigned ERVs as promising therapeutic targets in cancer treatment. Future studies should focus on better understanding the role of ERVs as possible tumor antigens and the underlying mechanism for epigenetically modulating TME for immunotherapy.^37^

In conclusion, decitabine sensitized chemotherapy and orchestrated with R-CHOP in *TP53*^mut^ DLBCL via SUV39H1-H3K9me3-ERVs axis, underlines a new anti-tumor mechanism of decitabine through modulating histone methylation. These findings further defined the clinical rationale for ERVs as potential epigenetic therapeutic targets to reprogram TME in treating *TP53*^mut^-driven cancers.

## Materials and methods

### Patients and study design

This study enrolled a total of 667 newly diagnosed DLBCL patients, with 576 in the R-CHOP cohort and the remaining patients in the DR-CHOP cohort (n = 91). Our previous phase I/II trial (NCT02951728) demonstrated a high complete response (100%) and durable remission in *TP53*^mut^ DLBCL upon DR-CHOP treatment; therefore, we conducted an extension cohort of DR-CHOP in *TP53*^mut^ DLBCL. Patients of the DR-CHOP cohort were from our previous phase I/II trial of DR-CHOP (NCT02951728) and its extended cohort, as well as those who received DR-CHOP in Guidance-01 (NCT04025593). After induction immunochemotherapy, 17 patients received ASCT, including 13 in the R-CHOP cohort (3 in complete remission [CR], 1 after R-CHOP and 10 in CR after relapse from R-CHOP) and 4 in the DR-CHOP cohort (2 in CR after DR-CHOP and 2 in CR after relapse from DR-CHOP). CAR-T was performed in 24 patients who either were refractory to or relapsed from treatment, including 20 patients in the R-CHOP cohort (8 with *TP53*^mut^ and 12 with *TP53*^wt^) and 4 patients in the DR-CHOP cohort (3 with *TP53*^mut^ and 1 with *TP53*^wt^). Histological diagnosis was established according to the World Health Organization classification,^40^ excluding mediastinal large B-cell lymphoma or primary central nervous system DLBCL. Treatment response was evaluated according to the International Workshop Criteria.^41^ The Hospital Review Board approved the study with informed consent obtained following the Declaration of Helsinki.

### Cell lines

B-lymphoma cell lines, SU-DHL-4 and OCI-LY-3, were obtained from American Type Culture Collection (Manassas, VA, USA). CRISPR/Cas9 system was used to delete the *TP53* gene in SU-DHL-4 (*TP53*^wt^) and OCI-LY3 (*TP53*^wt^) cell lines, and viral particles containing purified plasmids expressing GV392-U6-sgRNA-EF1a-Cas9-FLAG-P2A-puro were used to generate SU-DHL-4 *TP53*^-/-^ and OCI-LY-3 *TP53*^-/-^ cells. The stably transfected clones were selected by puromycin (10μg/μL). SU-DHL-4 *TP53*^-/-^ was then transfected with viral particles containing purified plasmids CV186-Cherry-puro-*TP53* ^p.R175H^, CV186-Cherry-puro-*TP53* ^p.R248Q^, CV186-Cherry-puro-*TP53* ^p.R273C^, as well as vector controls CV186-Cherry-puro-TP53-vector (MOI = 50) using Lipofectamine 3000 transfection reagents (Invitrogen, Shanghai, China) according to the manufacturer’s instruction. The stably transfected clones (Supplementary Fig. 12b) were selected by Cherry using BD FacsAria. Cell lines were exposed to decitabine (330 nM) alone, doxorubicin (200 nM) alone, or combined treatment (decitabine 330 nM for 5 days followed by doxorubicin 200 nM for 2 days based on clinically achievable concentrations).^42^ Cells were grown in RPMI-1640 medium with 10% heat-inactivated fetal bovine serum (FBS) added in a humidified atmosphere containing 5% CO_2_ at 37 °C.

### In vitro co-culture system

PBMCs were obtained from healthy donors through ficoll density gradient centrifugation, yielding a heterogeneous population consisting of myeloid and lymphoid cells, including ~15% B cells, 70% T cells, 5% monocytes, and 10% natural killer (NK) cells. The effector (E) to target (T) ratio refers to the proportion of PBMCs to lymphoma cells. In this study, the E:T ratio was set at 5:1, as recommended in previous research.^43^ PBMCs were grown in RPMI-1640 medium with 10% heat-inactivated fetal bovine serum (FBS) added in a humidified atmosphere containing 5% CO_2_ at 37 °C.

### DNA sequencing and viral genome detection

Genomic DNA was isolated from frozen tumor tissue using a Wizard® Genomic DNA Purification Kit (Promega, Wisconsin-Madison, USA) and from formalin-fixed paraffin-embedded (FFPE) tumor tissue using a GeneRead DNA FFPE Tissue Kit (Qiagen, Hilden, Germany) according to the manufacturer’s instructions. For 128 patients, whole genome sequencing (WGS) was performed on frozen tumor tissue. For 153 patients, whole exome sequencing (WES) was performed on frozen tumor tissue (*n* = 125) and FFPE tumor tissue quality-controlled by agarose gel electrophoresis (*n* = 28). Targeted sequencing was performed on formalin-fixed paraffin-embedded tumor samples of 386 patients with genomic DNA extracted using GeneRead DNA formalin-fixed paraffin-embedded Tissue Kit (Qiagen, Hilden, Germany). A targeted sequencing panel was assessed using a custom Sure Select library (Agilent Technologies), including 55 genes related to the pathogenesis of DLBCL according to literature, or associated with significant alternations in gene expression, as revealed by RNA sequencing.

### RNA sequencing

RNA sequencing was performed on frozen tumor samples of 280 patients with RNA extracted using the RNeasy MinElute Cleanup Kit (Qiagen, Dusseldorf, German). RNA-seq protocols are optimized for 1 μg of total RNA. RNA integrity was assessed using RNA 6000 Nano Kit (Agilent, California, USA) on Agilent 2100 Bioanalyzer (Agilent, California, USA) with an RNA Integrity Number (RIN) value ≥ 8. High-quality RNA showed a 28S rRNA band at 4.5 kb at twice the intensity of the 18S rRNA band at 1.9 kb. Then RNA-seq was performed using Illumina HiSeq 2000 (Illumina, California, USA) at 40–60 million 150 bp paired-end reads per sample depth.

Viral sequences were assessed and quantified by the Kraken 2.^27^ ERVs were assessed and quantified by RepeatMasker (hg19) based on a database downloaded from Repeating Elements. The ERVs’ expression was further assessed based on the mean expression of the top 4 ERVs.

### Bioinformatic analyses

Bioinformatic analyses were performed by r 4.0.3, using R package “sva” to remove the batch effect. Raw reads were normalized, and differentially expressed genes (DEGs) were obtained with R package “limma” (v3.38.3). The gene set enrichment analysis was performed using the R package ‘clusterProfiler’ (v3.10.1). GO enrichment analysis and data visualization. The methylation score was assessed by single-sample gene set enrichment analysis (ssGSEA) in the GSVA R package (v1.45.0) based on hallmark genes for methylation processes downloaded from the Molecular Signatures Database (MSigDB).^28^

### Interferon assessment

Serum specimens of DLBCL patients were collected before (day 0) and after one cycle of DR-CHOP (day 21). Culture supernatants of cell lines and lysed PDX samples were collected before and after decitabine and doxorubicin exposition. Concentrations of interferons in serum and culture supernatant were measured using interferon-α ELISA kit (cat. no. 70-EK199-96, MultiSciences Biotechnology), interferon-β ELISA kit (cat. no. 70-EK1236-96, MultiSciences Biotechnology) and interferon-γ ELISA kit (cat. no. 70-EK180-96, MultiSciences Biotechnology) according to manufacturer’s protocols. Cytokine concentrations were determined by measuring optical density using a microplate reader at 450/570 nm.

### Flow cytometry

To detect the percentage of immune cells, co‐cultured cells were stained with anti-CD3 (612940, BD Pharmingen, San Diego, USA), anti-CD4 (624298, BD Pharmingen, San Diego, USA), anti-CD8 (563919, BD Pharmingen, San Diego, USA). Data were analyzed using flowjo software (Becton Dickinson, Franklin Lakes, USA).

### Quantitative real-time PCR

Total mRNA was isolated using TRIzol reagent (Invitrogen, Shanghai, China). Complementary DNA was synthesized using HiScript III RT SuperMix for qPCR ( + gDNA wiper) (R323-01, Vazyme, Nanjing, China). Quantitative real-time PCR (qRT-PCR) was performed by ChamQ Universal SYBR qPCR Master Mix (Q711-03, Vazyme, Nanjing, China) with primers against ERVL-E, MER21C, HERV16, HERVK11, SUV39H1, E2F1, DNMT1, and GAPDH was used as an endogenous control. Relative expressions were calculated by the method of _ΔΔ_CT. The primer sequences of the above genes are listed in Supplementary Table 5.

### Western blot

Western blot was conducted following the procedures described previously.^44^ SU-DHL-4 and OCI-LY-3 were isolated and lysed in a lysis buffer (200 μl) (Sigma Aldrich, Shanghai, China). Protein lysates (10 μg) were electrophoresed on 10% sodium dodecyl sulfate-polyacrylamide gel electrophoresis (SDS-PAGE) and transferred to nitrocellulose membranes (BioRad, Hercules, CA, USA). Membranes were blocked for non-specific binding using 5% non-fat dried milk and left overnight at 4 °C rocking at low speed with H3K9me3 (ab8898, Abcam, Cambridge, MA, USA), SUV39H1 (PA5-87177, Invitrogen, Cambridge, MA, USA), DNMT1 (sc-271729, Santa Cruz, Dallas, Texas, USA), GAPDH (3683S, Cell Signaling Technology, Danvers, MA, USA), Histone H3 (ab1791, Cambridge, MA, USA) as primary antibodies. A horseradish peroxidase-conjugated antibody was employed for secondary detection. The immunocomplexes were visualized using an enhanced chemiluminescence detection kit.

### ChIP-seq

Nuclear extracts were derived from a total of 2 × 10^7^ cells per sample. Immunoprecipitation was performed using a rabbit anti-human H3K9me3 antibody (ab8898, Abcam, Cambridge, MA, USA), while a normal IgG (3900, Cell Signaling Technologies) was utilized as a negative control. The DNA libraries underwent 15 cycles of amplification and were subsequently subjected to sequencing using the Illumina NovaSeq 6000 platform with paired-end 2 × 150 sequencing mode. To ensure the acquisition of high-quality clean reads, raw reads underwent a filtration process that involved the removal of sequencing adapters, short reads (length < 35 bp), and low-quality reads using Cutadapt (v1.9.1)^45^ and Trimmomatic (v0.35).^46^ FastQC is then used to ensure high reads quality. Peak detection was performed using the MACS (v2.1.1)^47^ peak finding algorithm with 0.01 set as the *p*-value cutoff. Annotation of peak sites to gene features was performed using the ChIPseeker R package.^48^

### Immunohistochemistry

Immunohistochemistry was performed on 5 μm-paraffin sections with an indirect immunoperoxidase method using antibodies against H3K9me3 (1:400) (ab8898, Abcam, Cambridge, MA, USA) and SUV39H1 (1:200) (PA5-87177, Invitrogen, Cambridge, MA, USA). H3K9me3 and SUV39H1 expression levels were assessed according to the percentage of positive cells: + denoted <25%; ++ denoted 25-49%; +++ denoted 50–74%; ++++ denoted 75–100%.

### Lentivirus construction, short hairpin RNAs and plasmids

ShRNA lentivirus of *SUV39H1* was constructed by Xitubio Biotech (Shanghai Xitubio Biotechnology Co., Ltd, Shanghai, China). ShRNA sequence was provided as follows: 5’-AGTCGAGTACCTGTGCGATTA-3’. Non-targeting shRNA sequence was provided as follows: 5’-CAACAAGATGAAGAGCACCAA-3’.

### Immunofluorescence assay

For the immunofluorescence assay, methanol-fixed cells were stained using antibodies against H3K9me3 (1:400) (ab8898, Abcam, Cambridge, MA, USA) and SUV39H1 (1:200) (PA5–87177, Invitrogen, Cambridge, MA, USA). Donkey Anti-Rabbit IgG H&L (Alexa Fluor® 647) (1:200) (ab150075, Abcam, Cambridge, MA, USA) and goat Anti-Rabbit IgG H&L (Alexa Fluor® 488) (1:200) (ab150077, Abcam, Cambridge, MA, USA) were used as secondary antibodies.

### Lentivirus packaging and transfection

The coding sequence of the human *E2F1* gene (GenBank Accession: NM_005225.3) with C-terminal 3×flag was synthesized and cloned into a pCMV vector between HindIII and BamHI sites. The resulting plasmid was confirmed by Sanger sequencing. To overexpress *E2F1* in SU-DHL-4 and OCI-LY-3 cells, purified plasmid *E2F1*-over or vector were transfected into HEK-293T using lipofectamine 2000 (Invitrogen, Cambridge, MA, USA). The HEK-293T cell culture supernatant was condensed into a viral concentration of ~3 × 10^8^ transducing units/mL. The lentiviral particles were incubated with SU-DHL-4 and OCI-LY-3 cells, respectively, for 72 h. Stably transfected cells were generated through the introduction of GFP.

### Luciferase-reporter assay

The promoter region (−1500/ + 24) of the SUV39H1 gene was PCR-amplified from human genome DNA. The primer sequences were as follows: forward primer: 5’-CCTGAGCTCGCTAGCCTCGAGGAACATCTAACTGAATGACAGCCT-3’; reverse primer: 5’-CAGTACCGGATTGCCAAGCTTTCCCCACGGCTAACGACA −3’. The amplified 1.5 kb fragment was then cloned into pGL4.14 vector between XhoI and HindIII sites. The resulting plasmid was confirmed by Sanger sequencing. Luciferase-reporter assay was conducted using Duo-Lite Luciferase Assay System (DD1205-01, Vazyme, Nanjing, China) according to the manufacturer’s protocols.

### Methylation-specific PCR

Cells that underwent treatment with decitabine and/or doxorubicin were collected for DNA isolation utilizing a genomic DNA kit as per the instruction provided by the manufacturer (A1120, Promega, Wisconsin-Madison, USA), and then the DNA samples were treated using DNA Bisulfite Conversion Kit (DP215-02, Tiangen, China) and methylation-specific PCR Kit (EM101, Tiangen, China). The methylation and unmethylation primers utilized in this study are detailed in the Supplementary Table 5. The methylation-specific PCR products (200 ng) were electrophoresed on 2% agarose gels and visualized through ethidium bromide staining.

### Patient-derived tumor xenografted model

Four-week-old female NSG and NOD-SCID mice were obtained from Shanghai Laboratory Animal Center (Shanghai, China) to establish and maintain PDX models. Heterotopic PDX models were generated as previously described.^49^ Decitabine and doxorubicin, either alone or in combination, were applied to PDX models with a low passage number (P3-P5) to preserve the genetic integrity of the parental tumors. Tumor volumes were calculated as 0.5 × a × b^2^, where ‘a’ is the length and ‘b’ is the width. Treatments were started after the tumor became about 0.5 × 0.5 cm on the surface (day 0). The dose and administration schedule were as follows: decitabine pretreatment (10 mg/m^2^, 5 days), followed by doxorubicin (0.6 mg/kg every other day) four times, while the control group was untreated. Tumor-bearing mice were then euthanized by CO_2_ asphyxiation. Animals were used according to the ARRIVE guidelines and the protocols approved by Shanghai Rui Jin Hospital Animal Care and Use Committee.

### Statistics

All statistical analyses were performed in GraphPad Prism software (GraphPad Software, San Diego, CA, version 7.0), SPSS v23.0, and R v3.6.1 Survival Estimates were calculated using the Kaplan-Meier method, and survival curves were compared by the log-rank test. Univariate hazard estimate was generated with Cox proportional hazards models. Variables with a *p*-value <0.2 were included in the multivariate logistic regression analysis.^26^ Unpaired *t*-test with or without Welch’s correction was used to compare different groups, and Pearson rank correlation was used to calculate the correlation between the two groups. Fisher’s exact tests were applied to compare non-ordinal categorical variables. A two-sided *p*-value of <0.05 was considered statistically significant.

### Supplementary information

Supplemental material

## Data Availability

All the methods or reagents we used are accessible on the market. Sequencing data generated in this study have been deposited at the National Omics Data Encyclopedia (NODE, https://www.biosino.org/node/) under accession number OEP001143 and OEP004511.
