# Supplementary material for: Human endogenous retroviruses as epigenetic therapeutic targets in TP53-mutated diffuse large B-cell lymphoma
Source: Signal Transduct Target Ther. 2023 Oct 6;8:381. doi: 10.1038/s41392-023-01626-x (PMC10556001; doi:10.1038/s41392-023-01626-x)
Supplement: Supplementary file 1 — Supplemental material [file 41392_2023_1626_MOESM1_ESM.docx]

Supplementary Materials for

Human endogenous retroviruses as epigenetic therapeutic targets in *TP53*-mutated diffuse large B-cell lymphoma

Ying Fang^*^, Mu-Chen Zhang^*^, Yang He^*^, Chen Li, Hai Fang, Peng-Peng Xu, Shu Cheng, Yan Zhao, Yan Feng, Qian Liu, Li Wang^†^, Wei-Li Zhao^†^

**†** Correspondence to: Wei-Li Zhao, e-mail: zhao.weili@yahoo.com, Li Wang, email: wl11194@rjh.com.cn.

**This PDF file includes:**

Supplementary Figures (Figure S1-S12)

Supplementary Tables (Table S1-S5)


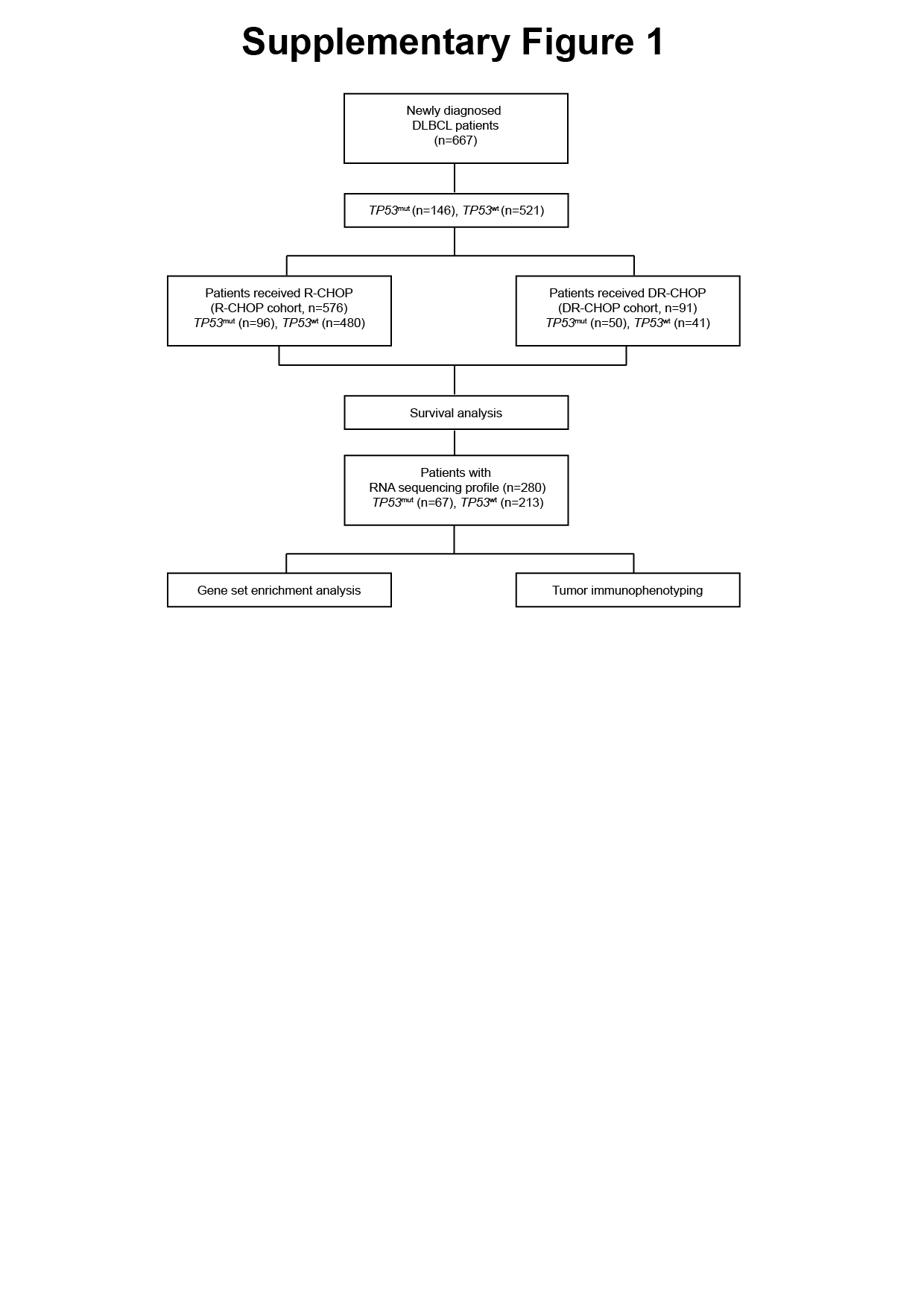


Supplementary Figure 1.

Flowchart of the patient selection and methods.


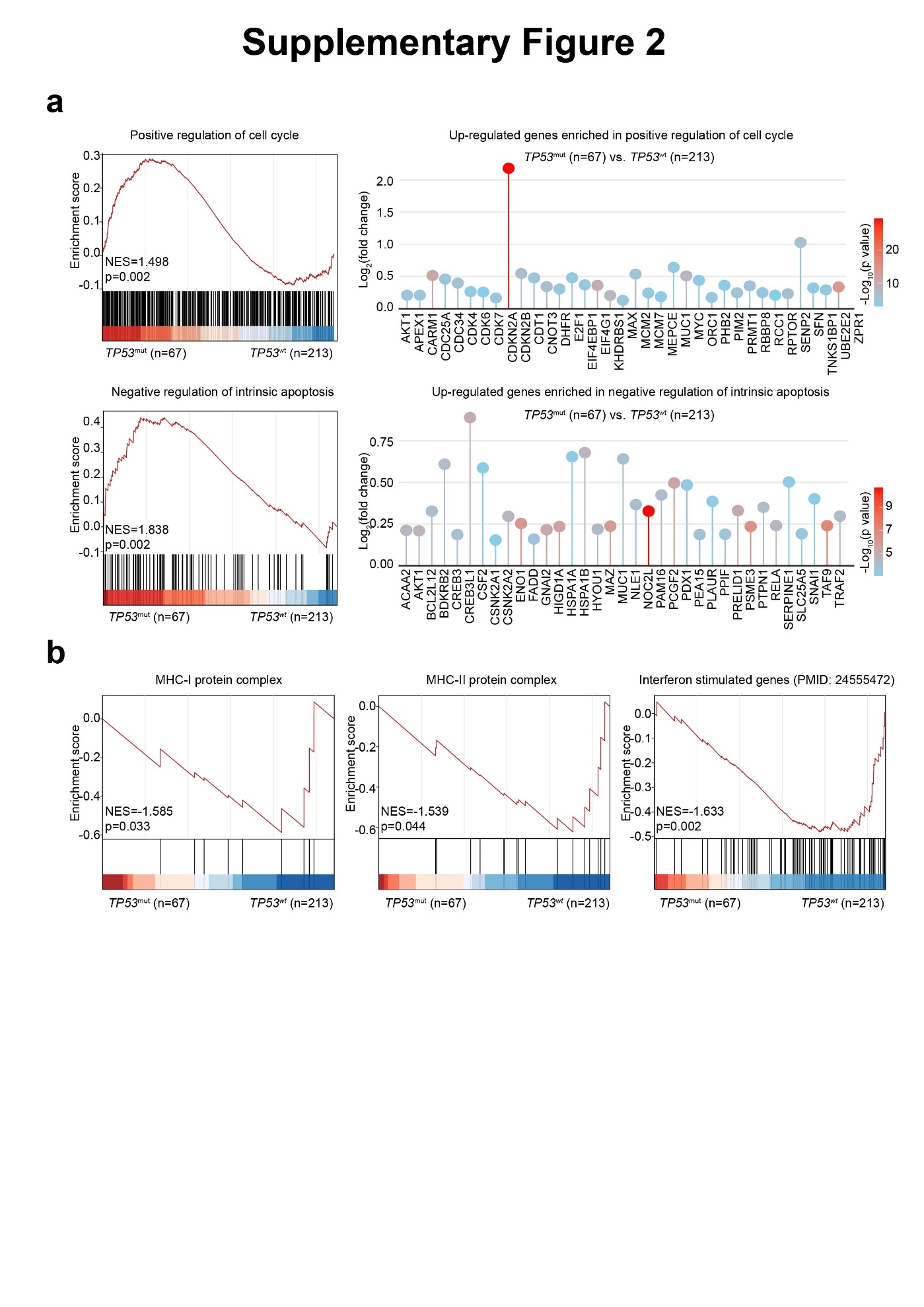


Supplementary Figure 2.

Pathway and genetic alterations in *TP53*^mut^ DLBCL.

(a) Up-regulated pathways and genes in positive cell cycle regulation and negative regulation of intrinsic apoptosis pathway in *TP53*^mut^ DLBCL patients.

(b) The enriched genes involved in MHC-I, MHC-II protein complex, and IFN-stimulated genes in *TP53*^mut^ DLBCL, as compared to *TP53*^wt^ DLBCL patients.


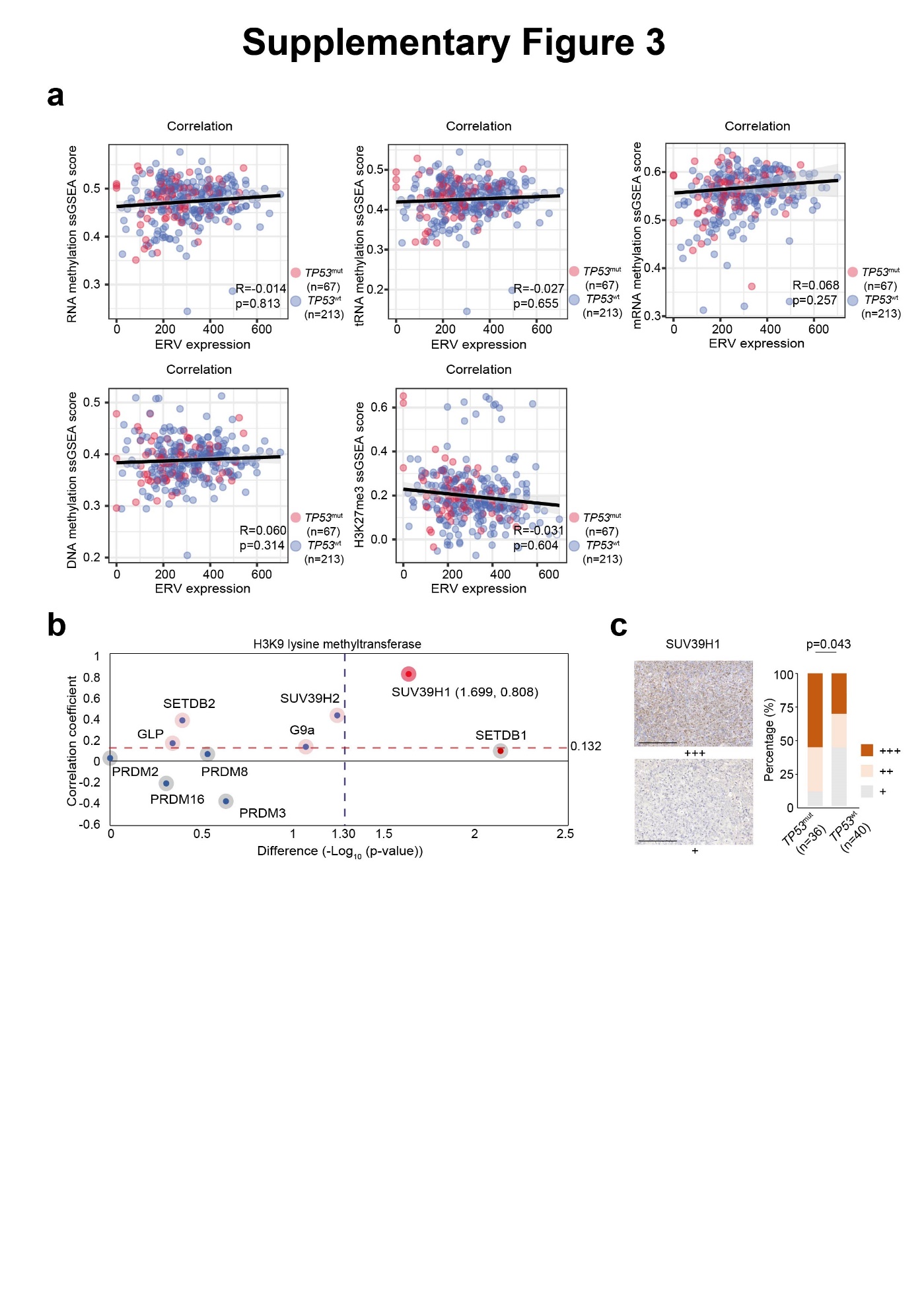


Supplementary Figure 3.

Correlations of methylation processes with ERV expression and correlations of histone methyltransferase expression with H3K9me3 score.

(a) No significant correlation observed among other methylation processes, such as RNA, tRNA, mRNA, DNA, and H3K27me3 methylation and ERV expression levels.

(b) The dot plot of differentially expressed methyltransferases of H3K9 between *TP53*^mut^ and *TP53*^wt^ DLBCL. X-axis represents the statistical differences of genes between *TP53*^mut^ and *TP53*^wt^ DLBCL, and the y-axis represents the correlation coefficient between the expression of genes and H3K9me3 score. The dashed lines correspond to p=0.05.

(c) Increased SUV39H1 expression revealed by immunohistochemistry staining on *TP53*^mut^ tumors. Left panel, representative immunohistochemistry staining images for SUV39H1. Right panel, proportion of SUV39H1 expression. Scale bars, 100 μm. Expression levels are scored based on the percentage of positive cells: +, <25%; ++, 25-49%; +++, 50-74%.


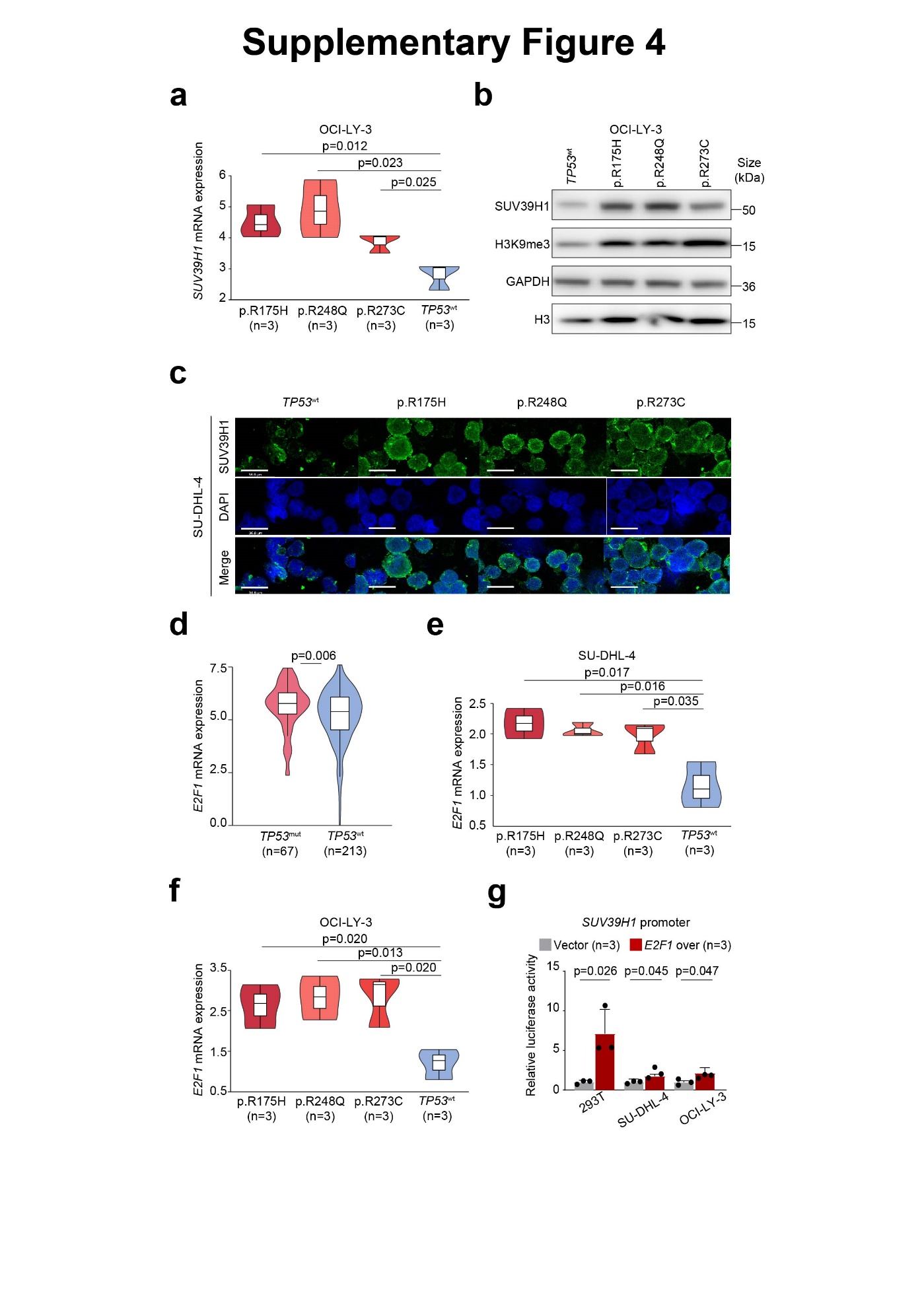


Supplementary Figure 4.

Expressions of SUV39H1 and E2F1 in *TP53*^mut^ cell lines.

(a-b) Increased mRNA expression of *SUV39H1* (a) and protein expression of SUV39H1 and H3K9me3 (b) in *TP53*^mut^ OCI-LY-3 cell line.

(c) Increased protein expression of SUV39H1 in *TP53*^mut^ SU-DHL-4 cell line by immunofluorescence staining. Scale bars, 40 μm.

(d-f) Increased mRNA expression of *E2F1* in *TP53*^mut^ DLBCL patients according to RNA-seq (d), *TP53*^mut^ SU-DHL-4 cell line by qRT-PCR (e), and *TP53*^mut^ OCI-LY-3 cell line by qRT-PCR (f).

(g) Relative luciferase activity of *SUV39H1* promoter in indicated cells transfected with vector or *E2F1*-overexpressed plasmid.


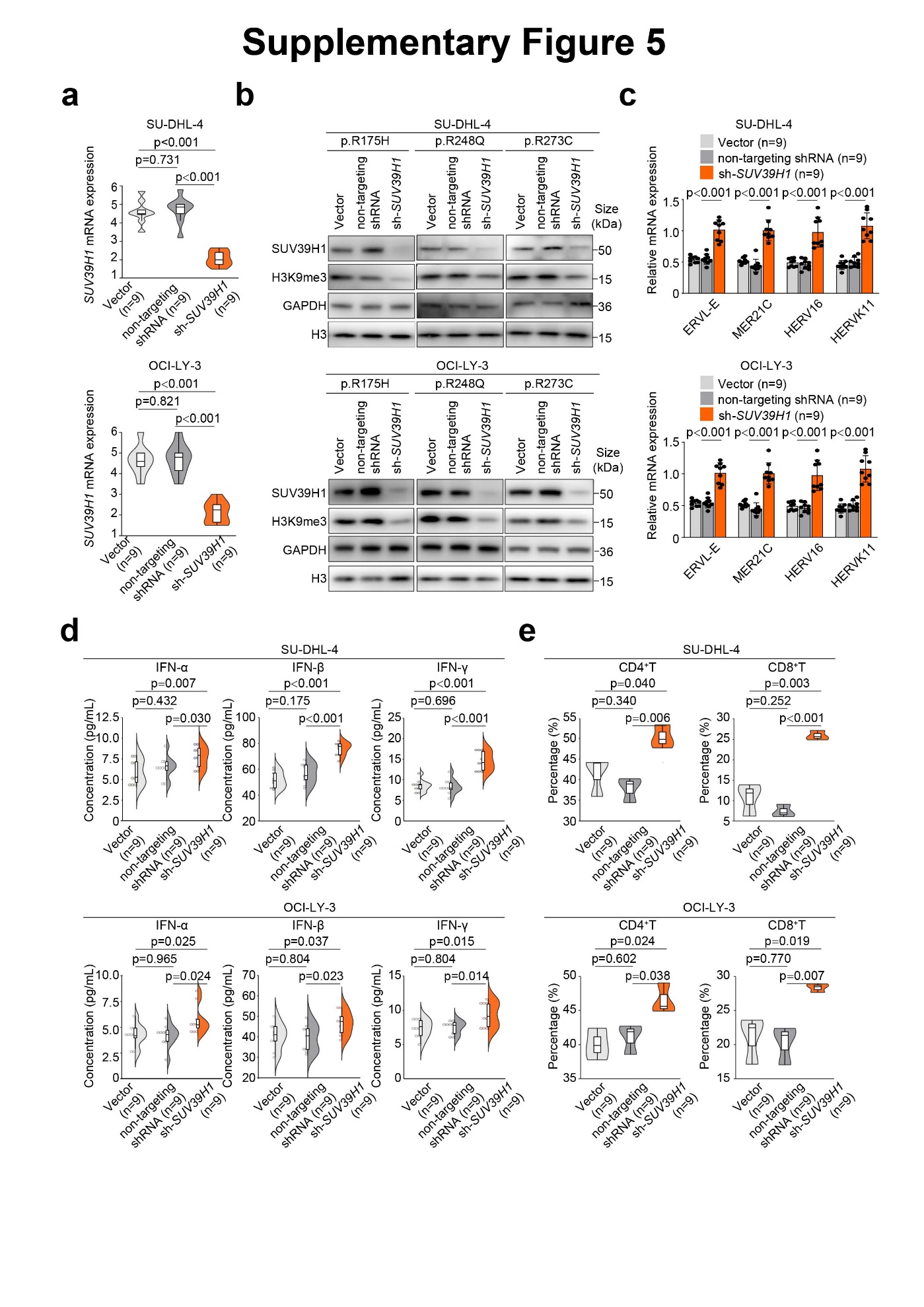


Supplementary Figure 5.

Knock-down of *SUV39H1* in *TP53*^mut^ cell lines.

(a-c) *SUV39H1* mRNA expression (a), SUV39H1 and H3K9me3 protein expression (b), and ERV expression (c) in *TP53*^mut^ SU-DHL-4 and OCI-LY-3 cell lines with knock-down of *SUV39H1*. Vector and non-targeting shRNA are used as control.

(d-e) IFN productions (d), percentages of CD3^+^CD4^+^T and CD3^+^CD8^+^T cells (e) in *TP53*^mut^ SU-DHL-4 and OCI-LY-3 cell lines with knock-down of *SUV39H1*.


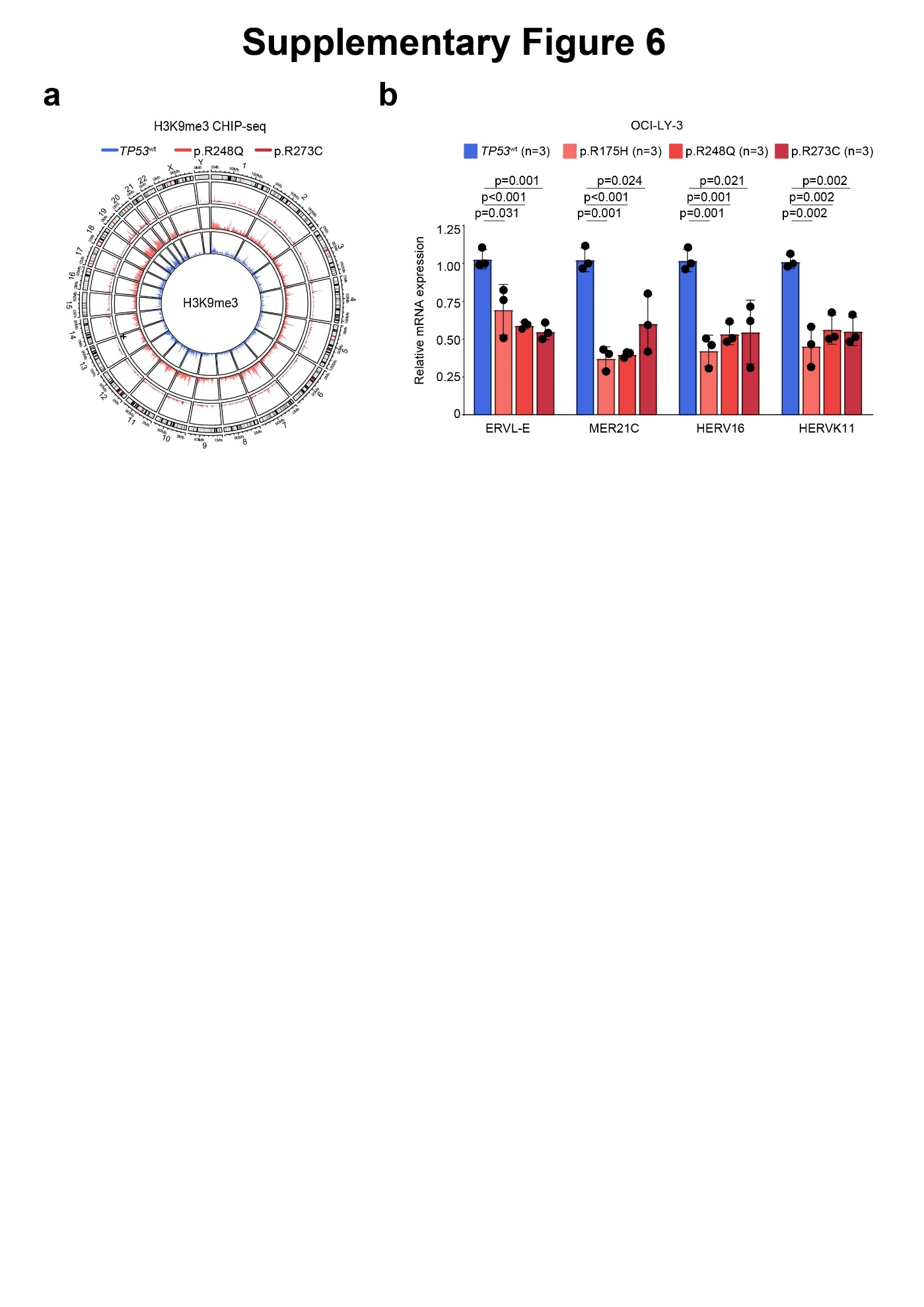


Supplementary Figure 6.

H3K9me3 enrichment and ERV expression in *TP53*^mut^ DLBCL.

(a) Distribution of H3K9me3 on the whole genome in *TP53*^mut^ p.R248Q, p.R273C or *TP53*^wt^ SU-DHL-4 cell line.

(b) Expression of ERV in *TP53*^mut^ or *TP53*^wt^ OCI-LY-3 cell line.


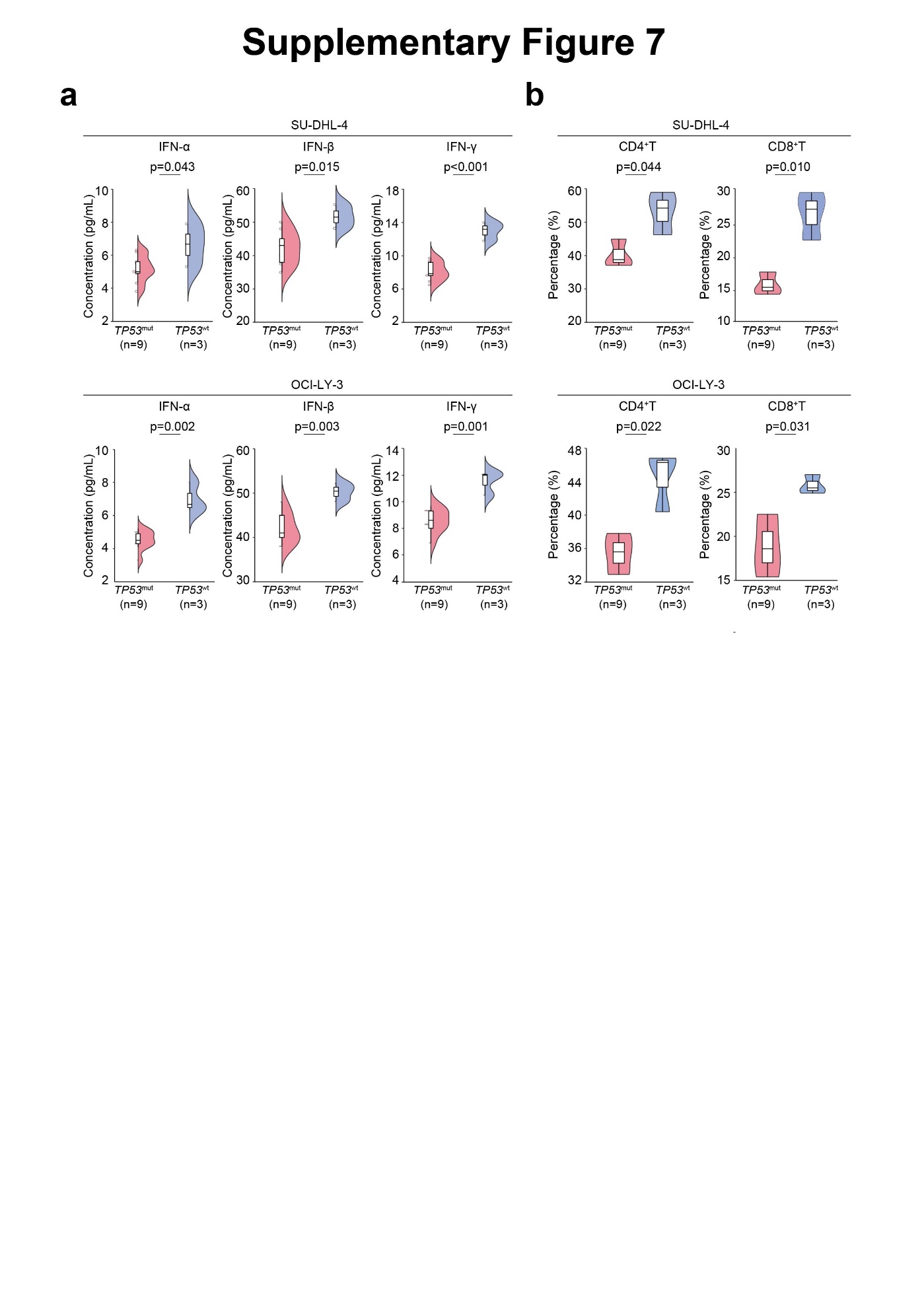


Supplementary Figure 7.

IFN production and T cell activation in *TP53*^mut^ cell lines.

(a) IFN production in *TP53*^mut^ or *TP53*^wt^ SU-DHL-4 and OCI-LY-3 cell lines.

(b) CD4^+^T and CD8^+^T cell proportions in co-culture of *TP53*^mut^ or *TP53*^wt^ SU-DHL-4 and OCI-LY-3 cell lines with PBMCs.


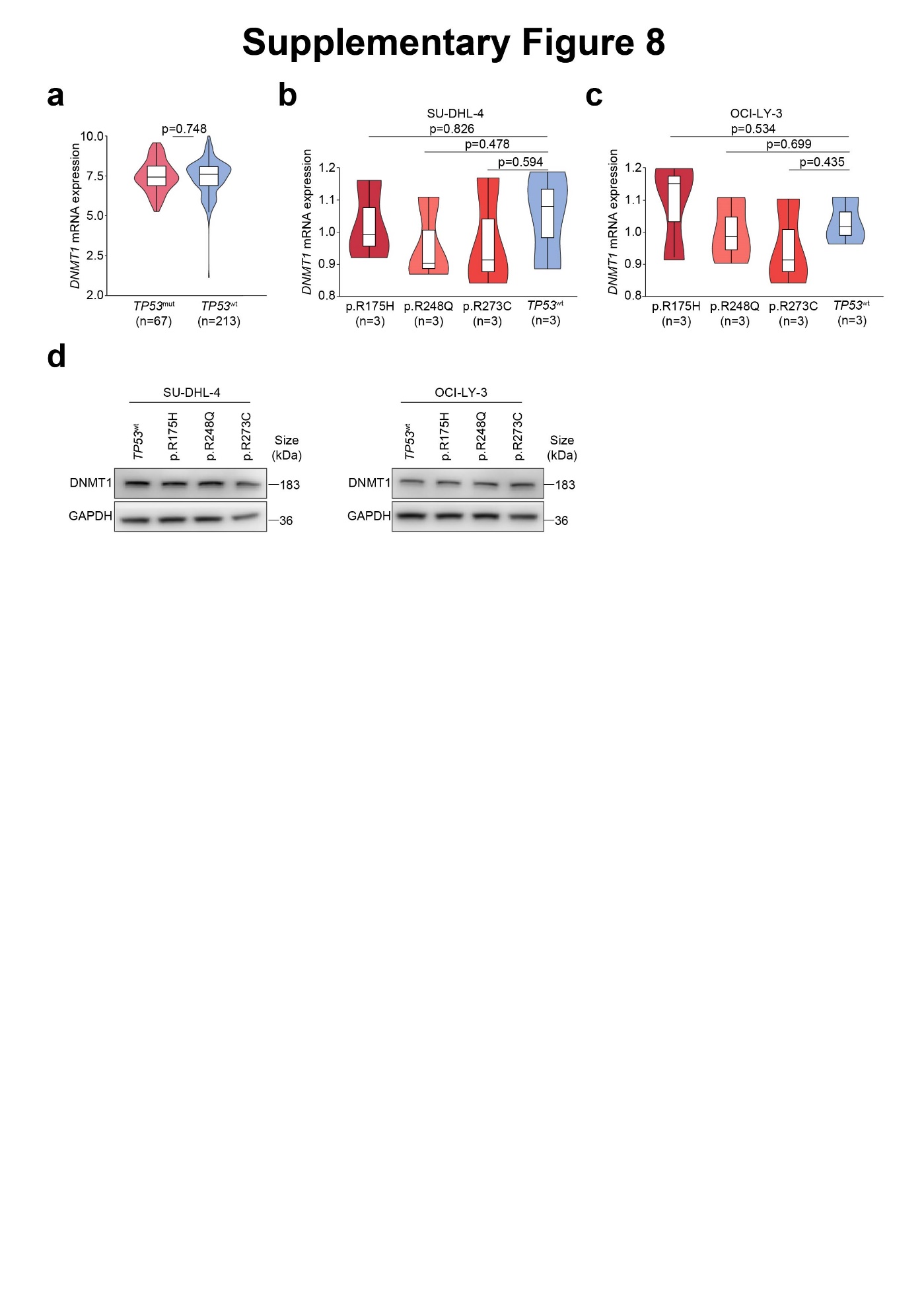


Supplementary Figure 8.

Expression of DNMT1 in *TP53*^mut^ DLBCL.

(a-c) *DNMT1* mRNA expression in *TP53*^mut^ DLBCL patients according to RNA-seq (a), *TP53*^mut^ SU-DHL-4 cell line by qRT-PCR (b), and *TP53*^mut^ OCI-LY-3 cell line by qRT-PCR (c).

(d) DNMT1 protein expression in *TP53*^mut^ SU-DHL-4 and OCI-LY-3 cell lines by western blot.


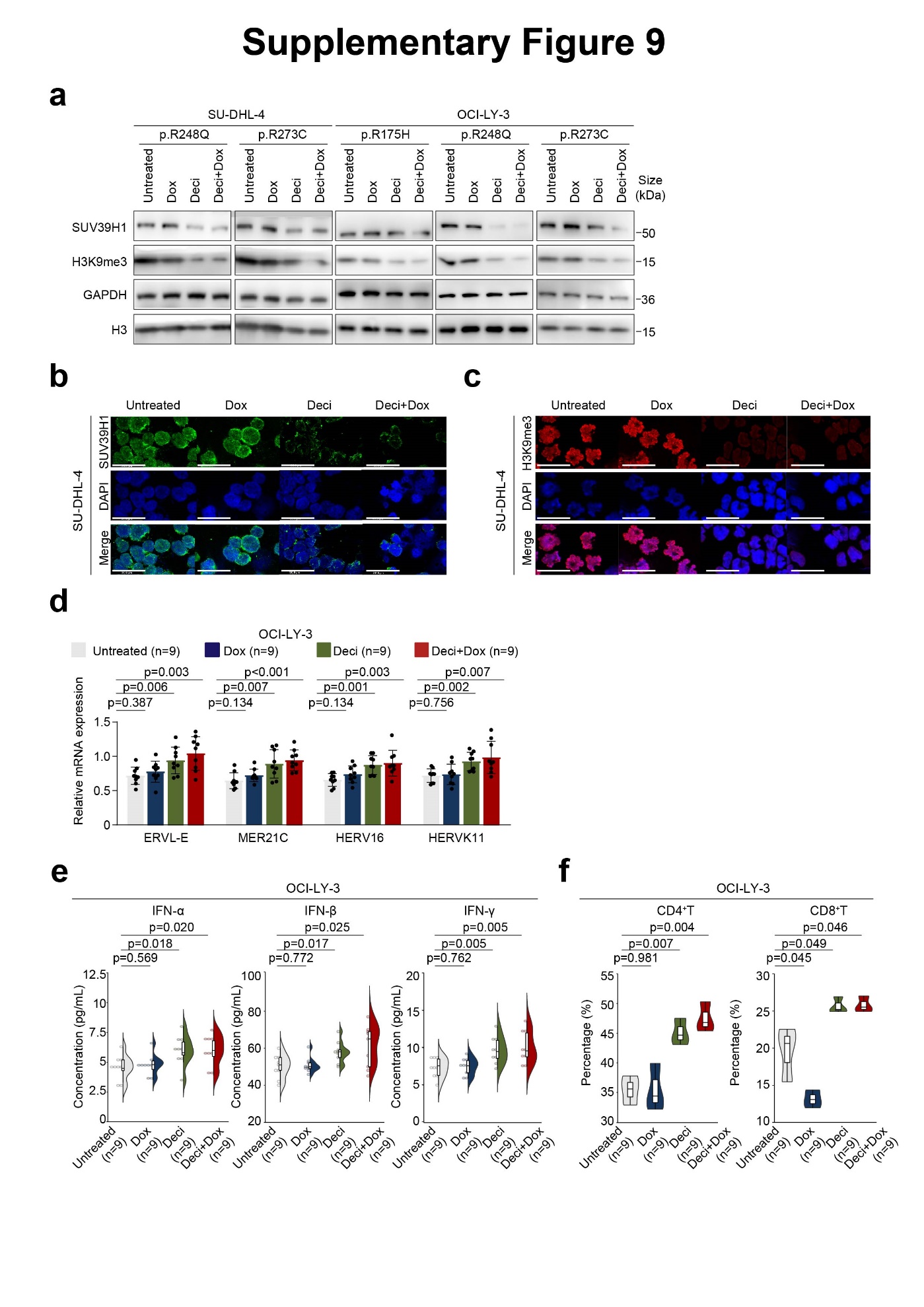


Supplementary Figure 9.

Expression of SUV39H1, H3K9me3, ERV, and IFN upon decitabine and/or doxorubicin treatment.

(a) Expression of SUV39H1 and H3K9me3 in *TP53*^mut^ SU-DHL-4 and OCI-LY-3 cell lines with decitabine, doxorubicin, alone or in combination.

(b-c) Expression of SUV39H1 (b) and H3K9me3 (c) in *TP53*^mut^ SU-DHL-4 cell line by immunofluorescence staining. Scale bars, 40 μm.

(d-f) ERV expression (d), IFN production (e), as well as percentages of CD4^+^T and CD8^+^T cells (f) in *TP53*^mut^ OCI-LY-3 cell line upon indicated treatment group.


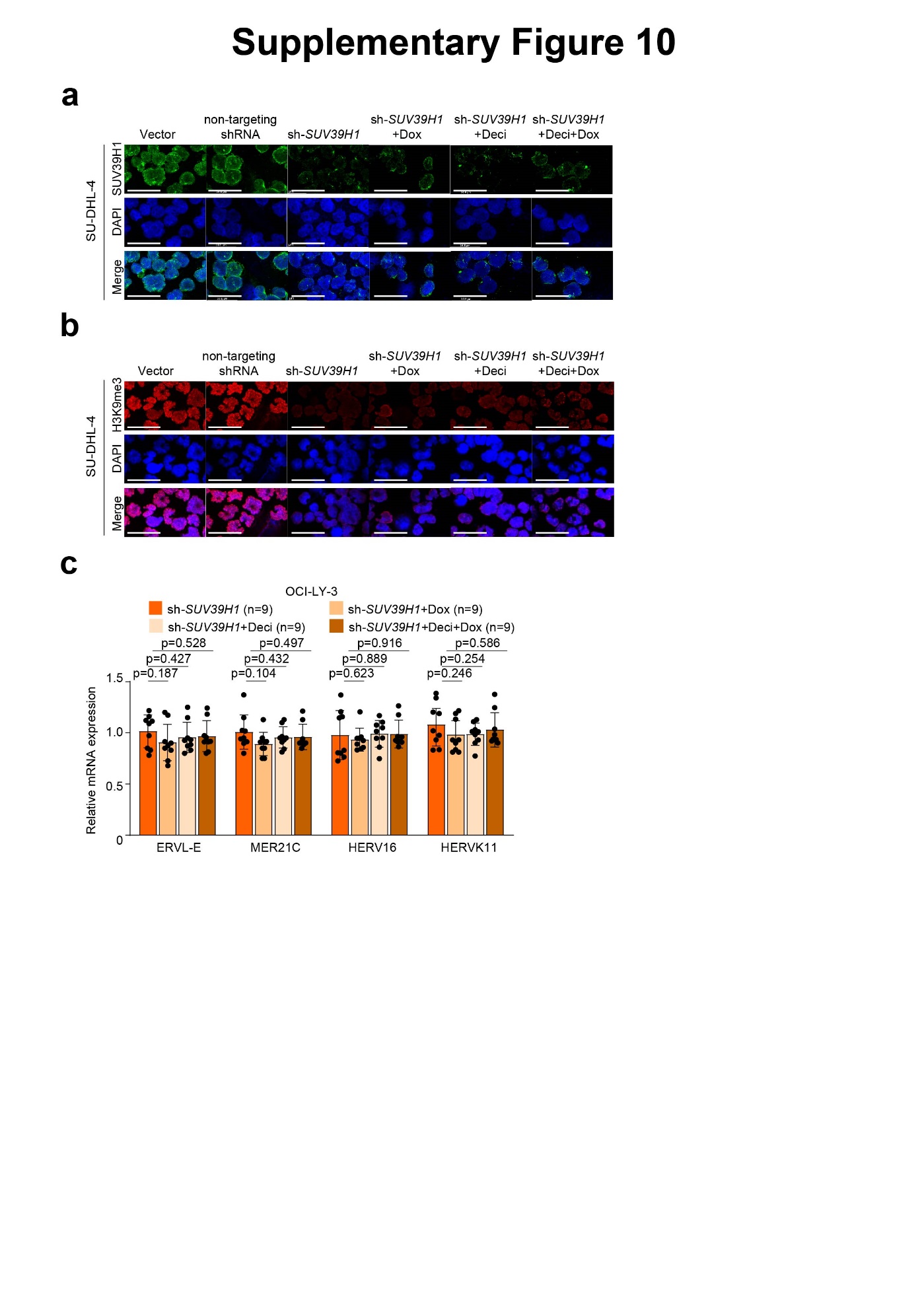


Supplementary Figure 10.

SUV39H1 and ERV expression in *TP53*^mut^ cell lines with knock-down of *SUV39H1* after decitabine and/or doxorubicin treatment.

(a-b) Expression of SUV39H1 (a) and H3K9me3 (b) in *TP53*^mut^ SU-DHL-4 cell line with knock-down of *SUV39H1* by immunofluorescence staining upon indicated treatment group. Scale bars, 40 μm.

(c) No difference of ERV expression in *SUV39H1* knock-down OCI-LY-3 cell line upon indicated treatment group.


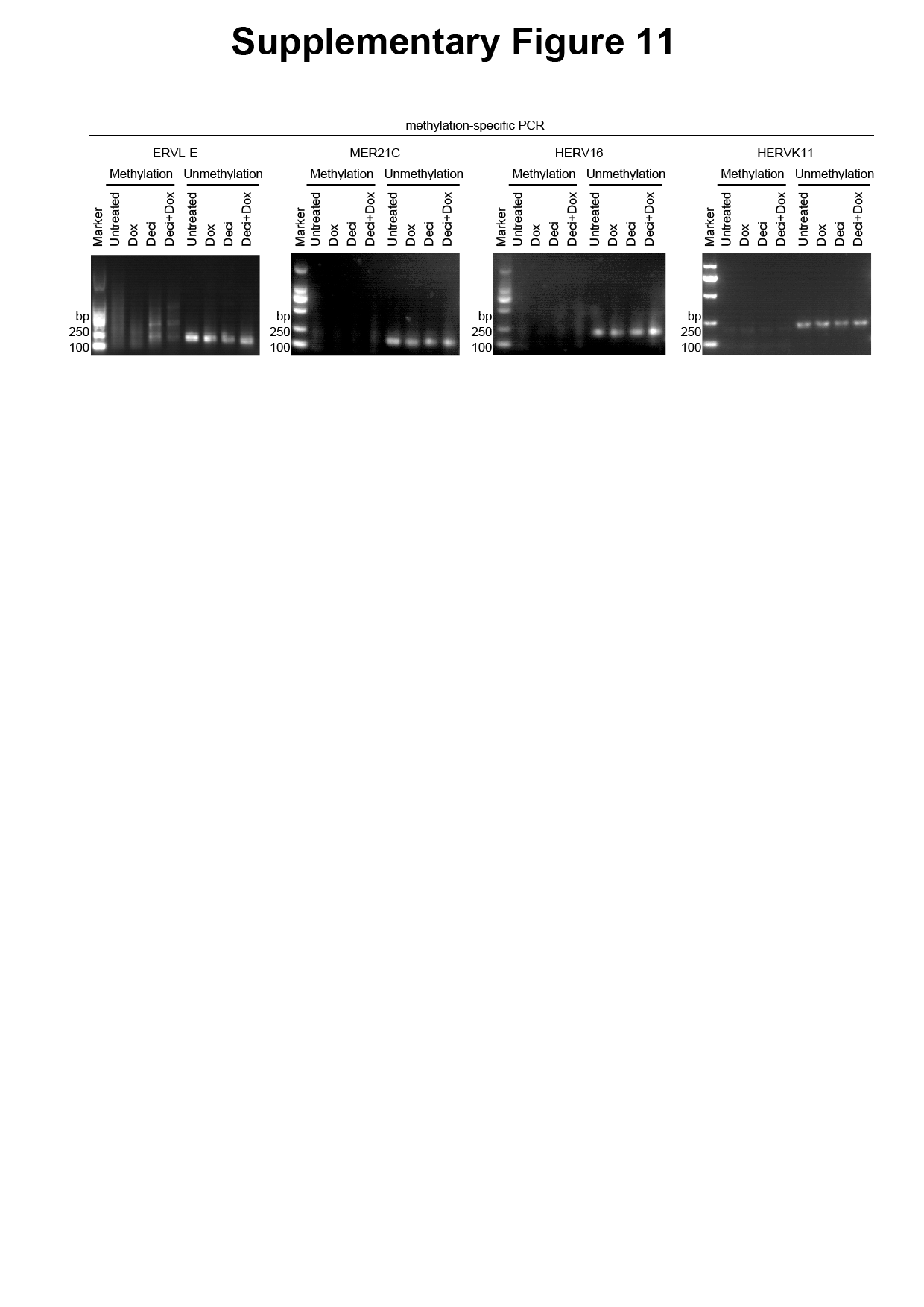


Supplementary Figure 11.

Methylation-specific PCR of ERV after decitabine and/or doxorubicin treatment.


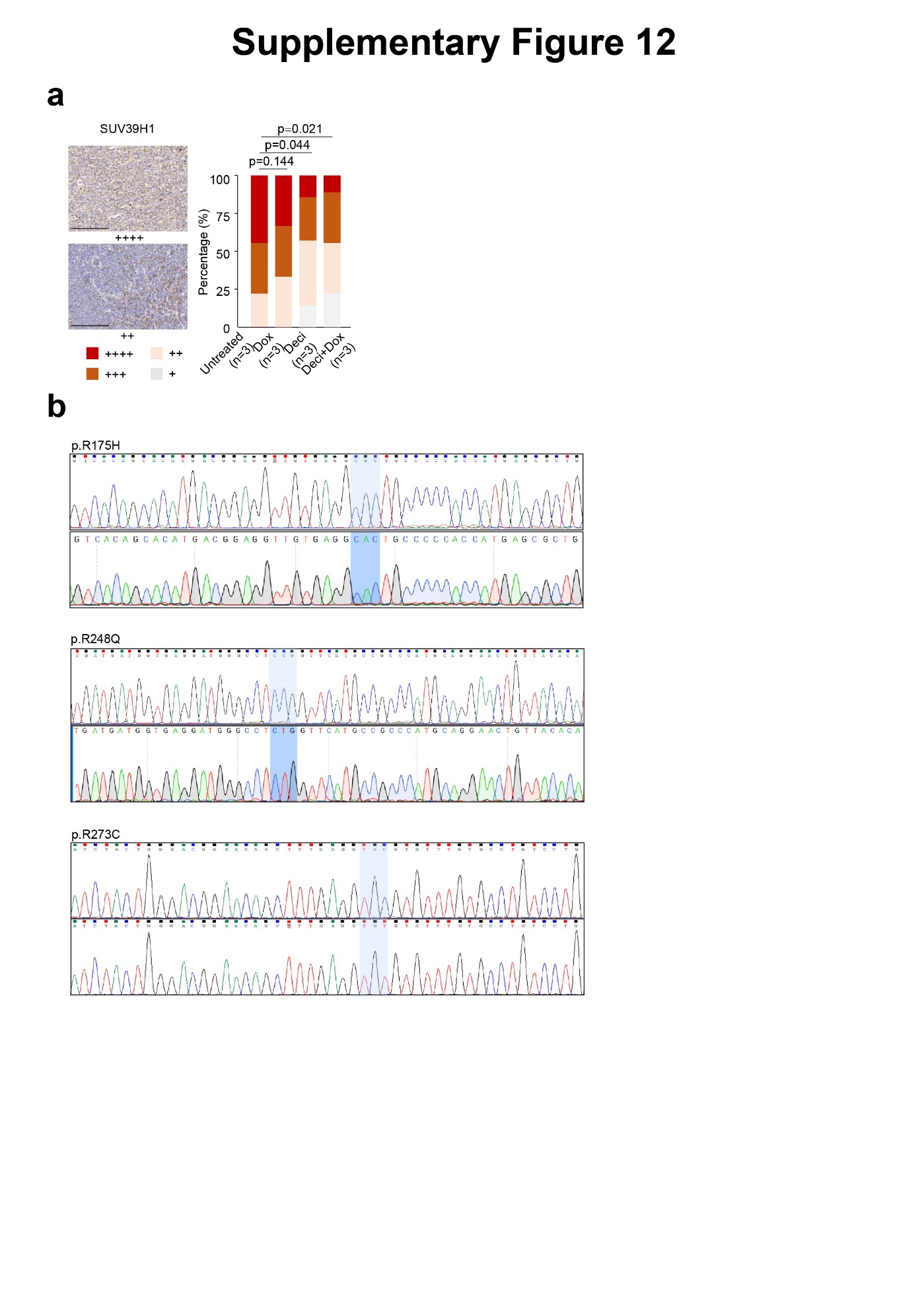


Supplementary Figure 12.

SUV39H1 expression in PDX model and Sanger sequencing of the mutant site in 3 stably transfected *TP53*^mut^ cell lines.

(a) Increased SUV39H1 expression revealed by immunohistochemistry staining on *TP53*^mut^ PDX tumors. Left panel, representative immunohistochemistry staining images for SUV39H1. Right panel, proportion of SUV39H1 expression. Scale bars, 100 μm. Expression levels were assessed according to the percentage of positive cells: + denoted < 25%; ++ denoted 25-49%; +++ denoted 50-74%; ++++ denoted 75-100%.

(b) Sanger sequencing of the mutant site in 3 stably transfected *TP53*^mut^ cell lines.

Supplementary Table 1.

Clinical characteristics of 667 DLBCL patients.

|  | *TP53*^mut^ n=146 | | *TP53*^wt^ n=521 | |  |
| --- | --- | --- | --- | --- | --- |
|  | n | % | n | % | p value |
| Age |  |  |  |  |  |
| Median (range) | 53.5 （17-84） | | 59 （16-85） | |  |
| ≤60 | 80 | 54.8% | 269 | 51.6% | 0.499 |
| >60 | 66 | 45.2% | 252 | 48.4% |  |
| Sex |  |  |  |  |  |
| Male | 70 | 47.9% | 288 | 55.3% | 0.116 |
| Female | 76 | 52.1% | 233 | 44.7% |  |
| ECOG |  |  |  |  |  |
| 0-1 | 135 | 92.5% | 476 | 91.4% | 0.671 |
| ≥2 | 11 | 7.5% | 45 | 8.6% |  |
| Stage |  |  |  |  |  |
| I/II | 77 | 52.7% | 281 | 53.9% | 0.798 |
| III/IV | 69 | 47.3% | 240 | 46.1% |  |
| LDH |  |  |  |  |  |
| Normal | 62 | 42.5% | 282 | 54.1% | 0.013 |
| Elevated | 84 | 57.5% | 239 | 45.9% |  |
| Extranodal sites |  |  |  |  |  |
| 0-1 | 108 | 74.0% | 381 | 73.1% | 0.839 |
| ≥2 | 38 | 26.0% | 140 | 26.9% |  |
| IPI |  |  |  |  |  |
| 0-2 | 100 | 68.5% | 374 | 71.8% | 0.438 |
| 3-5 | 46 | 31.5% | 147 | 28.2% |  |

Supplementary Table 2.

Multivariate analysis of factors associated with progression-free survival and overall survival in R-CHOP-treated DLBCL patients.

|  | Progression-free survival | | | Overall survival | | |
| --- | --- | --- | --- | --- | --- | --- |
|  | HR | 95%CI | p value | HR | 95%CI | p value |
| Age |  |  |  |  |  |  |
| ≤60 |  |  |  |  |  |  |
| >60 | 1.186 | 0.894-1.572 | 0.238 | 2.429 | 1.668-3.538 | <0.001 |
| Sex |  |  |  |  |  |  |
| Male |  |  |  |  |  |  |
| Female | / | / | / | 0.653 | 0.454-0.941 | 0.022 |
| ECOG performance status |  |  |  |  |  |  |
| 0-1 |  |  |  |  |  |  |
| ≥2 | 1.429 | 0.953-2.145 | 0.084 | 1.570 | 0.965-2.553 | 0.069 |
| Ann Arbor stage |  |  |  |  |  |  |
| I-II |  |  |  |  |  |  |
| III-IV | 3.057 | 2.161-4.326 | <0.001 | 2.592 | 1.655-4.061 | <0.001 |
| Serum LDH level |  |  |  |  |  |  |
| Normal |  |  |  |  |  |  |
| Elevated | 1.782 | 1.304-2.436 | <0.001 | 2.131 | 1.402-3.240 | <0.001 |
| Extranodal sites |  |  |  |  |  |  |
| 0-1 |  |  |  |  |  |  |
| ≥2 | 0.953 | 0.688-1.319 | 0.772 | 1.049 | 0.698-1.577 | 0.817 |
| *TP53* |  |  |  |  |  |  |
| wild-type |  |  |  |  |  |  |
| mutant | 1.895 | 1.376-2.610 | <0.001 | 2.191 | 1.448-3.317 | <0.001 |

Supplementary Table 3.

Multivariate analysis of factors associated with progression-free survival and overall survival in DR-CHOP-treated DLBCL patients.

|  | Progression-free survival | | | Overall survival | | |
| --- | --- | --- | --- | --- | --- | --- |
|  | HR | 95%CI | p value | HR | 95%CI | p value |
| Age |  |  |  |  |  |  |
| ≤60 |  |  |  |  |  |  |
| >60 | / | / | / | / | / | / |
| Sex |  |  |  |  |  |  |
| Male |  |  |  |  |  |  |
| Female | / | / | / | 0.225 | 0.045-1.132 | 0.070 |
| ECOG performance status |  |  |  |  |  |  |
| 0-1 |  |  |  |  |  |  |
| ≥2 | 3.007 | 1.068-8.462 | 0.037 | 6.184 | 1.458-26.237 | 0.013 |
| Ann Arbor stage |  |  |  |  |  |  |
| I-II |  |  |  |  |  |  |
| III-IV | 2.281 | 0.664-7.835 | 0.190 | / | / | / |
| Serum LDH level |  |  |  |  |  |  |
| Normal |  |  |  |  |  |  |
| Elevated | 1.719 | 0.485-6.096 | 0.402 | / | / | / |
| Extranodal sites |  |  |  |  |  |  |
| 0-1 |  |  |  |  |  |  |
| ≥2 | / | / | / | / | / | / |
| *TP53* |  |  |  |  |  |  |
| wild-type |  |  |  |  |  |  |
| mutant | / | / | / | / | / | / |

Supplementary Table 4.

Top 10 virus genera identified in DLBCL patients.

|  | *TP53*^mut^ n=67 | | | | | | | *TP53*^wt^ n=213 | | | |  | |
| --- | --- | --- | --- | --- | --- | --- | --- | --- | --- | --- | --- | --- | --- |
|  | | | | Total reads | Mean | Min | Max | Total reads | Mean | Min | Max | | p value |
| ERV | | | | |  |  |  |  |  |  |  | |  |
|  | | 17605 | | | 258.9 | 0 | 524 | 71723 | 338.3 | 21 | 1495 | | 0.001 |
| Lymphocryptovirus | | | | | |  |  |  |  |  |  | |  |
|  | | | 6508 | | 95.7 | 0 | 5223 | 28409 | 134.0 | 0 | 10288 | | 0.743 |
| Gammaretrovirus | | | | |  |  |  |  |  |  |  | |  |
|  | | | 1089 | | 16.0 | 0 | 343 | 20813 | 98.2 | 0 | 13362 | | 0.502 |
| Pahexavirus | | | | |  |  |  |  |  |  |  | |  |
|  | | | 528 | | 7.8 | 0 | 480 | 81 | 0.4 | 0 | 18 | | 0.066 |
| Gamaleyavirus | | | | |  |  |  |  |  |  |  | |  |
|  | | | 445 | | 6.5 | 0 | 199 | 2595 | 12.2 | 0 | 431 | | 0.430 |
| Andhravirus | | | | |  |  |  |  |  |  |  | |  |
|  | | | 421 | | 6.2 | 0 | 178 | 352 | 1.7 | 0 | 206 | | 0.120 |
| Cornellvirus | | | | | | |  |  |  |  |  | |  |
|  | | | 169 | | 2.5 | 0 | 73 | 737 | 3.5 | 0 | 163 | | 0.742 |
| Betabaculovirus | | | | |  |  |  |  |  |  |  | |  |
|  | | 71 | | | 1.0 | 0 | 51 | 455 | 2.1 | 0 | 84 | | 0.375 |
| Pandoravirus | | | | |  |  |  |  |  |  |  | |  |
|  | | | 68 | | 1.0 | 0 | 45 | 234 | 1.1 | 0 | 42 | | 0.879 |
| Seunavirus | | | | |  |  |  |  |  |  |  | |  |
|  | | | 25 | | 0.4 | 0 | 17 | 222 | 1.0 | 0 | 40 | | 0.297 |

Supplementary Table 5.

Primer sequences used in real-time quantitative reverse transcription-PCR and methylation-specific PCR.

| Gene | Forward | Reverse |
| --- | --- | --- |
| *ERVL-E* | 5′-TCTCTATTGATGCCTTTATGAGT-3′ | 5′-AGCATTGTGCTGTTTGTGAT-3′ |
| *MER21C* | 5′-GGAGCTTCCTGATTGGCAGA-3′ | 5′-ATGTAGGGTGGCAAGCACTG-3′ |
| *HERV16* | 5′-CACCAGAAGGTCACCAGATA-3′ | 5′-  CTGTTGGGGAGTCCAGTTCT-3′ |
| *HERVK11* | 5′-GGCTTGGTCCACAGATACAC-3′ | 5′-TGCCTTGAATAGAGTGACCA-3′ |
| *SUV39H1* | 5′-CCTGCCCTCGGTATCTCTAAG-3′ | 5′-ATATCCACGCCATTTCACCAG-3′ |
| *E2F1* | 5′-ACGCTATGAGACCTCACTGAA-3′ | 5′-TCCTGGGTCAACCCCTCAAG-3′ |
| *DNMT1* | 5′-AGGCGGCTCAAAGATTTGGAA-3′ | 5′-GCAGAAATTCGTGCAAGAGATTC-3′ |
| *GAPDH* | 5′-GGAGCGAGATCCCTCCAAAAT-3′ | 5′-GGCTGTTGTCATACTTCTCATGG-3′ |
| Methylation  *ERVL-E* | 5′-CTTTATGAGTTCTTTCGTAAGACAG-3′ | 5′-CATTGTGCTGTTTGTGATTAGTAG-3′ |
| Unmethylation  *ERVL-E* | 5′-ATGAGTTTTTTTGTAAGATAGATGTGA-3′ | 5′-TACAAATTACAACAATTCTCCTTTC-3′ |
| Methylation  *MER21C* | 5′-TGCTAGAAGGGTGATGTGCC-3′ | 5′-GGTTTGACAATTCACTGGAACA-3′ |
| Unmethylation  *MER21C* | 5′-TTGATATGTTAGAAGGGTGATGTGT-3′ | 5′-AACAATTCACTAAAACAACTCACAA-3′ |
| Methylation  *HERV16* | 5′-CCCAGAGGACACGTCATTTA-3′ | 5′-CTCTGCTTCTGGGAAACCTA-3′ |
| Unmethylation  *HERV16* | 5′-GGTTGGTGAGAGGTATATTTGTATT-3′ | 5′-AAATCACCACCTAACCTCTACTTCT-3′ |
| Methylation  *HERVK11* | 5′-TCTGTCTCTGATGAGCACGC-3′ | 5′-GTGCCCACAGAATAGCTTTG-3′ |
| Unmethylation  *HERVK11* | 5′-TTGAAAAATTTGTTTTTGATGAGTA-3′ | 5′-CCAATTTACTTAATATCCATAACCC-3′ |
